# Peripheral inflammatory immune response differs among sporadic and familial Parkinson’s disease

**DOI:** 10.1038/s41531-023-00457-5

**Published:** 2023-01-31

**Authors:** Laura Muñoz-Delgado, Daniel Macías-García, María Teresa Periñán, Silvia Jesús, Astrid D. Adarmes-Gómez, Marta Bonilla Toribio, Dolores Buiza Rueda, María del Valle Jiménez-Jaraba, Belén Benítez Zamora, Rafael Díaz Belloso, Sergio García-Díaz, Miguel Martín-Bórnez, Rocío Pineda Sánchez, Fátima Carrillo, Pilar Gómez-Garre, Pablo Mir

**Affiliations:** 1 Unidad de Trastornos del Movimiento, Servicio de Neurología y Neurofisiología Clínica, Instituto de Biomedicina de Sevilla, Hospital Universitario Virgen del Rocío/CSIC/Universidad de Sevilla, Seville, Spain; 2 Centro de Investigación Biomédica en Red sobre Enfermedades Neurodegenerativas (CIBERNED), Madrid, Spain; 3 Departamento de Medicina, Facultad de Medicina, Universidad de Sevilla, Seville, Spain

**Keywords:** Parkinson's disease, Inflammation

## Abstract

Peripheral inflammatory immune responses are thought to play a major role in the pathogenesis of Parkinson’s disease (PD). The neutrophil-to-lymphocyte ratio (NLR), a biomarker of systemic inflammation, has been reported to be higher in patients with PD than in healthy controls (HCs). The present study was aimed at determining if the peripheral inflammatory immune response could be influenced by the genetic background of patients with PD. We included a discovery cohort with 222 patients with PD (132 sporadic PD, 44 *LRRK2*-associated PD (with p.G2019S and p.R1441G variants), and 46 *GBA*-associated PD), as well as 299 HCs. Demographic and clinical data were recorded. Leukocytes and their subpopulations, and the NLR were measured in peripheral blood. Multivariate lineal regression and post-hoc tests were applied to determine the differences among the groups. Subsequently, a replication study using the Parkinson’s Progression Markers Initiative cohort was performed which included 401 patients with PD (281 sPD patients, 66 *LRRK2*-PD patients, 54 *GBA*-PD patients) and a group of 174 HCs. Patients with sporadic PD and *GBA*-associated PD showed a significantly lower lymphocyte count, a non-significantly higher neutrophil count and a significantly higher NLR than HCs. The peripheral inflammatory immune response of patients with *LRRK2*-associated PD did not differ from HCs. Our study supports the involvement of a peripheral inflammatory immune response in the pathophysiology of sPD and *GBA*-associated PD. However, this inflammatory response was not found in *LRRK2*-associated PD, probably reflecting different pathogenic inflammatory mechanisms.

## Introduction

Parkinson’s disease (PD) is the second most common neurodegenerative disease whose etiopathogenesis is still unknown. PD is considered to result from a complex interplay between aging, genetic susceptibility, and exposure to environmental factors^1^. A wide variety of hypotheses have been put forward to explain the cause of neurodegeneration in PD, including inflammation. Both central and peripheral inflammatory immune responses have been documented in PD and they are thought to play a major role in the initiation and progression of neurodegeneration in PD^2–8^. Ageing itself, the major risk factor for PD, is also associated with a low-grade inflammatory status of the immune system.

To begin with, studies have shown that neurodegeneration in PD is accompanied by microglial activation, but also the substantia nigra of patients with PD has been shown to be infiltrated by T lymphocytes^2,3^. Moreover, a brain-periphery interaction is suggested to be present in PD. Cytokines, which communicate and modulate peripheral and central immune compartments, seem to be dysregulated in the blood, cerebrospinal fluid and brains of patients with PD compared to healthy controls (HCs)^5^. Concerning the peripheral immune activation, leukocytes (which are the immune cells in peripheral blood) and their subpopulations have been described to be quantitatively and qualitatively altered in patients with PD^4^. A decreased absolute lymphocyte count and particularly an impairment in CD4 + T cells subpopulation have been consistently reported in the literature^7,9^. It has been hypothesised that this dysregulation in lymphocytes population may diminish their protective immune function and promote a pro-inflammatory environment that could contribute to neurodegeneration^7^. In fact, it has recently been reported that a lower lymphocyte count might be causally related to the subsequent development of PD^10^. Even so, there are discrepancies between findings in T lymphocytes populations dysregulation, which may be explained by the heterogeneous cohorts evaluated in the studies. On the other hand, a higher neutrophil count in patients with PD has been also described in many cohorts^9^. However, few studies have specifically addressed the role of neutrophils in PD etiopathogenesis and whether the alterations in this population are cause or consequence remains to be further studied. More recently, our group has also studied the peripheral immune response in patients with PD compared to HCs through the neutrophil-to-lymphocyte ratio (NLR), a well-established non-invasive indicator of the overall inflammatory status of the organism^11,12^. Our group reported that patients with PD had an altered peripheral immune response and a higher NLR compared with HCs, supporting the existence of an inflammatory systemic status in PD^13^.

Genetic factors should be also considered in the complex etiopathogenesis of PD. Several genes have been associated with the familial forms of PD, from variants in genes responsible for Mendelian forms of the disease (such as leucine-rich repeat kinase 2, *LRRK2*) to common risk *loci* that increase susceptibility for PD (such as heterozygous variants in glucocerebrosidase, *GBA*, gene)^1,14^. The identification of these genes have provided new insights into PD etiopathogenesis since they encode proteins involved in specific cellular pathways related to immunity and inflammation^15,16^. Although LRRK2’s function in cells is not completely understood, it is highly expressed in immune cells and regulates the immune responses^4,17^. In patients with PD, it has been described an increased expression of LRRK2 protein levels in immune cells, such as monocytes or lymphocytes^2,3,15^. Patients with *LRRK2*-associated PD (*LRRK2*-PD) may show a more pro-inflammatory cytokine profile compared to HCs, which has been also related to clinical severity^18^. Moreover, polymorphisms in the *LRRK2* gene have been linked to inflammatory and infectious diseases^14^. On the other hand, several studies highlight a link between *GBA* gene and inflammation in PD^19^. Early studies identified increased plasma levels of inflammatory markers and cytokines in *GBA*-associated PD (*GBA*-PD) compared to sporadic PD (sPD) patients and HCs^20^. Even so, there are controversial results between studies, probably due to differences between *GBA* variants or populations included.

It is worth mentioning that patients with mutations in these PD-associated genes have distinct clinical features and disease progression, which at the same time have been linked to different inflammatory profiles^14^. For example, patients with *LRRK2*-PD are usually considered to have a milder phenotype and to be less likely to develop cognitive impairment compared to sPD or *GBA*-PD^14^. At the same time, clinical features such as a more severe motor impairment or cognitive decline have been associated to a higher pro-inflammatory profile in patients with PD. Due to the complex genotype, phenotype, and peripheral immune responses described in patients with PD, it could be hypothesized that the different genetic background in patients with PD might be associated with an involvement of different inflammatory pathways.

Altogether, the existing data highlights the importance of studying the peripheral inflammatory immune response in PD patients. We hypothesized that the peripheral inflammatory immune response could be influenced by the genetic background of patients with PD. Subsequently, studies in PD genetic cohorts are needed to better delineate the role of the immune responses on the pathogenesis of the disease. The present study is aimed at determining whether there are differences in the peripheral inflammatory immune response between sPD and the main causes of familial PD (*LRRK2*-PD and *GBA*-PD).

## Results

### Characteristics of the whole case-control study

The demographic characteristics and the peripheral immune profile of the whole PD cohort and HCs from the discovery and the PPMI replication cohorts are summarized in Table 1.

**Table 1:** Demographic and clinical features, as peripheral immune profile of healthy controls and patients with Parkinson’s disease of the discovery cohort and the PPMI replication cohort.

	Discovery cohort			PPMI replication cohort		
	HCs (*n* = 299)	Patients with PD (*n* = 222)	*P* value	HCs (*n* = 173)	Patients with PD (*n* = 401)	*P* value
Age (y), mean ± SD	59.94 ± 15.31	62.84 ± 11.72	< 0.05^a^	59.8 ± 12.24	60.88 ± 10.09	0.27^a^
Sex (% males)	51.17	55.86	0.29^b^	64.37	62.30	0.64^b^
Age of onset (y), mean ± SD	–	54.60 ± 12.08	–	–	58.67 ± 10.25	–
Disease duration (y), mean ± SD	–	8.25 ± 5.75	–	–	2.57 ± 3.55	–
HY in OFF state, mean ± SD	–	2.59 ± 0.94	–	–	1.63 ± 0.53	–
UPDRS-III in OFF state, mean ± SD	–	–	–	1.20 ± 2.27	21.08 ± 9.12	<0.001^a^
MOCA, mean ± SD	–	–	–	28.24 ± 1.14	26.89 ± 2.55	<0.001^a^
LEDD, mean ± SD	–	827.51 ± 540.76	–	–	80.62 ± 218.07	–
Leukocyte count (x10^3^ cells/μL), mean ± SD	6.89 ± 1.7	6.81 ± 1.51	0.64^c^	6.16 ± 1.81	6.24 ± 1.53	0.58^c^
Lymphocytes (x10^3^ cells/μL), mean ± SD	2.15 ± 0.65	1.86 ± 0.6	<0.001^c^	1.85 ± 0.91	1.69 ± 0.52	<0.05^c^
Neutrophils (x10^3^ cells/μL), mean ± SD	3.92 ± 1.25	4.24 ± 1.27	<0.001^c^	3.67 ± 1.17	3.97 ± 1.27	<0.05^c^
Monocytes (x10^3^ cells/μL), mean ± SD	0.52 ± 0.36	0.46 ± 0.15	<0.05^c^	0.41 ± 0.16	0.38 ± 0.12	<0.05^c^
Eosinophils (x10^3^ cells/μL), mean ± SD	0.24 ± 0.36	0.18 ± 0.41	0.37^c^	0.17 ± 0.13	0.16 ± 0.13	0.12^c^
Basophils (x10^3^ cells/μL), mean ± SD	0.08 ± 0.54	0.03 ± 0.03	0.27^c^	0.05 ± 0.03	0.05 ± 0.03	0.49^c^
NLR, mean ± SD	1.98 ± 0.89	2.51 ± 1.13	<0.001^c^	2.18 ± 0.79	2.53 ± 1.11	<0.001^c^

*HC* healthy control, *PD* Parkinson’s disease, *n* total number of subjects, *y* years, *SD* standard deviation, *HY* Hoehn & Yahr stage, *UPDRS-III* Unified Parkinson’s Disease Rating Scale part III, *MOCA* Montreal Cognitive Assessment, *LEDD* levodopa equivalent daily dose, *NLR* neutrophil-to-lymphocyte ratio.

^a^Based on Welch two-sample *t* tests.

^b^Based on *χ*^2^ test.

^c^Based on multivariate linear regression, adjusting for age and sex.

In the discovery cohort, patients with PD were slightly older than HCs, without differences in sex ratio between groups. The mean age of onset was 54.60 ± 12.08 years, the mean disease duration of 8.25 ± 5.75 years and the mean Hoehn & Yahr (HY) stage in OFF state of 2.59 ± 0.94. Patients with PD had lower lymphocyte and monocyte counts than HCs, but a higher neutrophil count. The NLR was significantly higher in patients with PD than in HCs. The differences remained statistically significant after adjusting for age and sex.

In the PPMI replication cohort, there were no differences in age and sex between the whole PD cohort and HCs. The mean age of onset was 58.67 ± 10.25, and they had a shorter disease duration (2.57 ± 3.55 years) and a minor motor disease severity (in OFF state, HY stage of 1.63 ± 0.53 and MDS Unified Parkinson’s Disease Rating Scale part III (MDS-UPDRS-III) scale of 21.08 ± 9.12 points) compared to the discovery cohort. Lower lymphocyte and monocyte count as well as a higher neutrophil count were also found among the whole PD group compared to HCs. Patients with PD had a significantly higher NLR than HCs. The differences remained statistically significant after adjusting for age and sex.

### Parkinson’s disease cohorts’ study

#### Discovery cohort study

The demographic and clinical characteristics of the different PD cohorts (sPD, *LRRK2*-PD, and *GBA*-PD) and HCs from the discovery cohort are shown in Table 2. The sPD group was older than HCs. The *GBA*-PD group was younger and showed a lower age of PD onset than sPD and *LRRK2*-PD. sPD and *GBA*-PD had a predominance of males, whereas *LRRK2*-PD showed a higher proportion of females. There were no differences between groups either in the duration or severity of the disease according to HY stage. The levodopa equivalent daily dose (LEDD) was higher in the *LRRK2*-PD patients compared to sPD.

**Table 2:** Demographic and clinical data, and peripheral immune profile of healthy controls and Parkinson’s disease genetic cohorts of the discovery cohort.

	HCs (*n* = 299)	sPD (*n* = 132)	*LRRK2*-PD (*n* = 44)	*GBA*-PD (*n* = 46)	*F* value	*P* value
Age (y), mean ± SD	59.94 ± 15.13	65.05 ± 11.72	62.48 ± 11.07	56.87 ± 10.35	6.28	<0.001^a^
Sex (% males)	51.17	60.60	38.64	58.70	–	0.05^b^
Age of onset (y), mean ± SD	NA	56.95 ± 12.09	53.41 ± 12.09	48.96 ± 10.09	9.01	<0.001^a^
Disease duration (y), mean ± SD	NA	8.05 ± 5.54	9.11 ± 6.32	7.98 ± 5.80	0.62	0.54^a^
HY in OFF state, mean ± SD	NA	2.03 ± 0.80	2.17 ± 0.73	2.07 ± 0.65	0.54	0.58^a^
LEDD, mean ± SD	NA	756 ± 491.02	998.63 ± 554.05	867.51 ± 628.19	3.55	0.03^a^
Leukocyte count (x10^3^ cells/μL), mean ± SD	6.89 ± 1.7	6.78 ± 1.51	6.81 ± 1.51	6.92 ± 1.52	1.61	0.16^c^
Lymphocytes (x10^3^ cells/μL), mean ± SD	2.15 ± 0.65	1.79 ± 0.62	2.02 ± 0.51	1.9 ± 0.57	8.12	<0.001^c^
Neutrophils (x10^3^ cells/μL), mean ± SD	3.92 ± 1.25	4.26 ± 1.27	4.06 ± 1.17	4.33 ± 1.38	4.05	<0.005^c^
Monocytes (x10^3^ cells/μL), mean ± SD	0.52 ± 0.36	0.45 ± 0.13	0.49 ± 0.19	0.48 ± 0.14	2.26	<0.05^c^
Eosinophils (x10^3^ cells/μL), mean ± SD	0.24 ± 0.36	0.2 ± 0.52	0.16 ± 0.11	0.15 ± 0.11	0.77	0.57^c^
Basophils (x10^3^ cells/μL), mean ± SD	0.08 ± 0.54	0.02 ± 0.03	0.03 ± 0.03	0.03 ± 0.02	0.80	0.55^c^
NLR, mean ± SD	1.98 ± 0.89	2.63 ± 1.13	2.11 ± 0.76	2.55 ± 1.33	12.06	<0.001^c^

*HC* healthy control, *PD* Parkinson’s disease, *n* total number of subjects, *y* years, *SD* standard deviation, *HY* Hoehn & Yahr stage, *LEDD* levodopa equivalent daily dose, *NLR* neutrophil-to-lymphocyte ratio.

^a^Based on linear regression and analysis of covariance (ANCOVA).

^b^Based on *χ*^2^ test.

^c^Based on multivariate linear regression, adjusting for age and sex.

When we compared the leukocyte subpopulations among the different PD cohorts and HCs, we found statistically significant differences in lymphocytes [*F*(5,515) = 8.12, *p* < 0.001], neutrophils [*F*(5,515) = 4.05, *p* < 0.005], and monocytes (*F*(5,515) = 2.26, *p* < 0.05] counts and also in the NLR [*F*(5,515) = 12.06, *p* < 0.001], after adjusting for sex and age (Table 2).

**Fig. 1:**
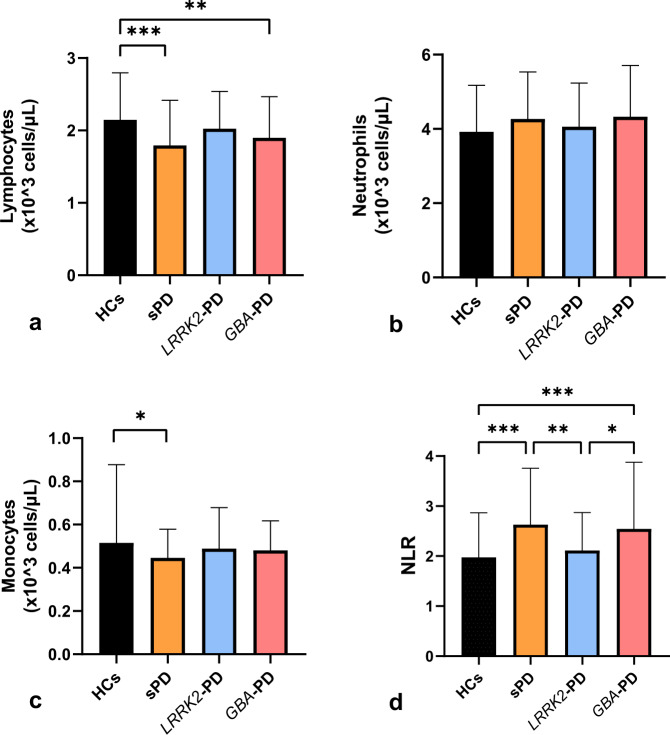
Peripheral inflammatory immune profile among sporadic and familial forms of Parkinson’s disease (PD) and healthy controls of the discovery cohort. *Multiple comparisons of lymphocytes (**a**), neutrophils (**b**), monocytes (**c**), and neutrophil-to-lymphocyte ratio (NLR) (**d**) among healthy controls (HCs), sporadic PD patients (sPD), *GBA*-associated PD patients (*GBA*-PD, and *LRRK2*-associated PD patients (*LRRK2*-PD). Numbers shown as means ± SD with multivariate lineal regression and post hoc testing for multiple comparisons as appropriate. ***p < 0.001, **p < 0.05, *p ≤ 0.1.

We subsequently accomplished multiple comparisons to determine the differences in the peripheral immune response between groups (Fig. 1a–d). The complete results of post hoc tests in the discovery cohort are described in Supplementary Information 1, part A, supplementary Tables 1, 3, 5, and 7.

The lymphocyte count was significantly lower in sPD and *GBA*-PD groups than in HCs. The neutrophil count was higher in the sPD and *GBA*-PD groups than in HCs, although these differences did not achieve statistical significance (*p* = 0.14 and *p* = 0.17, respectively). Finally, sPD and *GBA*-PD also had a significantly higher NLR than HCs. On the other hand, the *LRRK2*-PD group did not show significant differences in either the lymphocyte or the neutrophil count or in the NLR when compared to HCs.

Regarding the monocyte count, there was a marginal difference in the monocyte count between sPD and HCs (*p* = 0.05), without statistically significant differences among other groups. No differences were found in the leukocyte count nor in the basophils and eosinophils count.

Finally, since there was a higher female predominance in *LRRK2*-PD group, we also studied the peripheral inflammatory immune response stratified per sex. These results are shown in Supplementary Information 1, Part B, Supplementary Tables 9–15.

In both males and females’ cohorts, there were statistically significant differences in both lymphocyte counts and the NLR among PD groups and HCs. After multiple comparisons analyses, it was demonstrated that both lymphocyte counts and the NLR were higher in sPD in both males (*p* < 0.001 and *p* < 0.001, respectively) and females (*p* < 0.05 and *p* < 0.001, respectively) compared to HCs. Although a trend towards a lower lymphocyte count was observed in males in *GBA*-PD group compared to HCs, it was not statistically significant (*p* = 0.06). Concerning other blood cell counts, males showed a barely non-significant higher neutrophil count (*p* = 0.1) and a lower monocyte count (*p* = 0.06) in sPD compared to HCs, which were not observed in the female cohort. On the other hand, the *LRRK2*-PD group did not show significant differences in either the lymphocyte or the neutrophil count or in the NLR in sex-stratified PD groups when compared to HCs.

#### PPMI replication cohort study

Having found differences in the peripheral immune response among the different PD cohorts, we next tested our results for replication in an independent PPMI cohort. The detailed features of this cohort are shown in Table 3. No differences in age were found among groups. Whereas HCs, sPD, and *GBA*-PD had a male predominance, the *LRRK2*-PD showed a female predominance. Disease duration, motor, and cognitive impairment, as well as dopaminergic treatment were different among PD patients’ cohorts. Patients with *LRRK2*-PD showed a longer disease duration and a higher LEDD compared to the other groups. These differences are due to the different eligibility criteria in PPMI study for sPD and genetic cohorts (see Methods section).

**Table 3:** Demographic and clinical data, and peripheral immune response of healthy controls and Parkinson’s disease genetic cohorts of the PPMI replication cohort.

	HCs (*n* = 174)	sPD (*n* = 281)	*LRRK2*-PD (*n* = 66)	*GBA*-PD (*n* = 54)	*F* value	*P* value
Age (y), mean ± SD	59.8 ± 12.24	60.99 ± 10.12	61.64 ± 10.18	59.37 ± 9.9	0.87	0.46^a^
Sex (% males)	64.37	69.04	44.71	55.74	–	<0.001^b^
Age of onset (y), mean ± SD	–	59.54 ± 10.41	56.92 ± 10.57	56.97 ± 10.41	2.81	0.06^a^
Disease duration (y), mean ± SD	–	1.93 ± 1.84	4.63 ± 2.77	2.40 ± 2.15	45.64	<0.001^a^
HY in OFF state, mean ± SD	–	1.57 ± 0.50	1.95 ± 0.62	1.63 ± 0.53	11.84	<0.001^a^
MDS-UPDRS-III in OFF state, mean ± SD	–	21 ± 8.83	19.18 ± 8.23	23.57 ± 11.06	3.161	<0.05^a^
MOCA, mean ± SD	28.24 ± 1.14	27.11 ± 2.30	25.75 ± 3.38	27.11 ± 2.25	22.54	<0.001^a^
LEDD, mean ± SD	–	1.64 ± 20.25	428.45 ± 339.28	72.95 ± 187.97	204.13	<0.001^a^
Leukocyte count (x10^3^ cells/μL), mean ± SD	6.16 ± 1.81	6.18 ± 1.49	6.32 ± 1.79	6.48 ± 1.45	1.34	0.25^c^
Lymphocytes (x10^3^ cells/μL), mean ± SD	1.85 ± 0.91	1.66 ± 0.50	1.86 ± 0.61	1.66 ± 0.45	3.02	<0.05^c^
Neutrophils (x10^3^ cells/μL), mean ± SD	3.67 ± 1.17	3.93 ± 1.24	3.94 ± 1.41	4.22 ± 1.25	3.31	<0.05^c^
Monocytes (x10^3^ cells/μL), mean ± SD	0.41 ± 0.16	0.38 ± 0.13	0.37 ± 0.11	0.40 ± 0.12	8.81	<0.001^c^
Eosinophils (x10^3^ cells/μL), mean ± SD	0.17 ± 0.13	0.16 ± 0.14	0.15 ± 0.09	0.14 ± 0.09	1.24	0.29^c^
Basophils (x10^3^ cells/μL), mean ± SD	0.05 ± 0.03	0.05 ± 0.03	0.04 ± 0.02	0.05 ± 0.04	1.95	0.08^c^
NLR, mean ± SD	2.18 ± 0.79	2.56 ± 1.16	2.28 ± 0.92	2.68 ± 0.99	7.55	<0.001^c^

*HC* healthy control, *PD* Parkinson’s disease, *n* total number of subjects, *y* years, *SD* standard deviation, *HY* Hoehn & Yahr stage, *MDS-UPDRS-III* MDS Unified Parkinson’s disease rating part III scale, Montreal Cognitive Assessment, *LEDD* levodopa equivalent daily dose, *NLR* neutrophil-to-lymphocyte ratio.

^a^Based on linear regression and analysis of covariance (ANCOVA).

^b^Based on *χ*^2^ test.

^c^Based on multivariate linear regression, adjusting for age and sex.

Statistically significant differences were also found in lymphocytes [*F*(5568) = 3.02, *p* < 0.05], neutrophils [*F*(5568) = 3.31, *p* < 0.05], and monocytes (*F*(5576) = 8.81, *p* < 0.001] counts and also in the NLR [*F*(5564) = 7.55, *p* < 0.001] among PD cohorts and HCs, after adjusting for sex and age.

Again, we subsequently accomplished multiple comparisons to determine the differences in the peripheral immune response between groups. The complete results of post-hoc tests in the PPMI replication cohort are shown in Supplementary Information 1, Part A, Supplementary Tables 2, 4, 6, and 8.

A lower lymphocyte count was found in sPD and *GBA*-PD than in HCs, but it was only statistically significant in the sPD group. Both sPD and *GBA*-PD had higher neutrophil count than HCs, but it was only significant in the *GBA*-PD group. Finally, the NLR was significantly higher in both sPD and *GBA*-PD groups compared to HCs. Again, no differences in either the lymphocyte, or neutrophil counts or the NLR were found between the *LRRK2*-PD group and the HCs.

The monocyte count showed differences between groups. However, after performing multiple comparisons test, only a marginally significant difference was found between sPD and HC (*p* = 0.06), without differences between other groups. No differences were found in leukocytes, basophils, nor eosinophils between groups.

Finally, since there was also a female predominance in *LRRK2*-PD group in PPMI replication cohort, we also tested our results for replication in sex-stratified analyses. Detailed results are shown in Supplementary Information 1, Part A, Supplementary Tables 16–21.

Females showed lower lymphocyte and monocyte counts in sPD group compared to HCs, which was not observed in males. On the other hand, males showed a barely non-significant higher neutrophil count in sPD compared to HCs (*p* = 0.1), not observed in females. On top of that, in both male and female cohorts, there were statistically significant differences in the NLR among PD groups and HCs. After multiple comparisons analyses, the NLR was higher in sPD in males, and seemed to be higher in females compared to HCs, although the later did not reach an statistically significant level (*p* = 0.18). Again, the *LRRK2*-PD group did not show significant differences in either the lymphocyte or the neutrophil count or in the NLR in sex-stratified PD groups when compared to HCs.

#### Association of peripheral immune responses with PD clinical characteristics

Firstly, no correlation was found between disease duration and the peripheral inflammatory immune response studied in either the discovery cohort or the PPMI replication cohorts. Concerning motor impairment, no correlation was found between HY stage and the peripheral immune response in either the discovery or the PPMI replication cohort. Interestingly, a positive mild statistically significant correlation was found between the NLR, and the MDS-UPDRS-III scale in the whole PD cohort in the replication cohort. Moreover, this correlation seemed to be present regardless of the genetic status of patients with PD. Concerning cognitive impairment, no correlation was found between the MOCA score and the peripheral inflammatory immune response studied in the PPMI replication cohort. Detailed results are shown in the Supplementary Information 1, Part C.

## Discussion

This study specifically analysed if the peripheral inflammatory immune response could be different between sporadic and familial forms of PD. Our data indicated that sPD and *GBA*-PD had a significant lower lymphocyte count, a higher neutrophil count and a significantly higher NLR than HCs, supporting the presence of a peripheral inflammatory immune response. However, the *LRRK2*-PD group did not show differences in any leukocyte subpopulation nor in the NLR when compared to HCs. Remarkably, these results were replicated in the PPMI cohort.

Our results showed a higher NLR in the whole PD patients’ cohort when compared to HCs, which is in line with previous reports^13^. This ratio has been extensively used as a biomarker of peripheral inflammation. The NLR integrates information from two leukocyte subpopulations: neutrophils are associated with chronic inflammation^21^, and lymphocytes might represent the regulatory pathway.

The decreased absolute lymphocyte count in patients with PD is also consistent with the data reported in the literature^2,3,7,22^. There is growing evidence supporting the crucial involvement of T lymphocytes subpopulations in PD^20^^,23–25^. A meta-analysis including 21 case-control studies and 943 patients with PD supported that the numbers of CD3 + and CD4 + T lymphocytes were significantly decreased in PD^26^. In contrast with these results, other studies have found that patients with PD had an increase in the percentage of CD3 + and CD4 + T cells, whereas other groups did not find any significant differences compared to HCs^24^. Based on previous studies published, this decrease of CD4 + T cells could be due to an imbalance of specific subpopulations in peripheral blood responsible for different immune functions^4,7,24^. It has been observed an increased proportion of Th1 and Th17 cells (which are pro-inflammatory) and a decreased number of Th2 cells (which have anti-inflammatory functions), that could lead to a functional pro-inflammatory Th1-biased immune response in PD^27,28^. However, other groups have published an increase of Th17 and Th2 cells, without changes in Th1^22^. Diverse reports have been also reported on regulatory T cells, which are considered to have modulatory functions^22,27–31^. As discussed in detail below, the differences found in the lymphocyte counts between the different studies might be in part due to the genetic background. According to our results, the peripheral inflammatory immune response seems to differ between sporadic and some genetic forms of the disease, particularly in *LRRK2*-PD. Altogether, these findings could suggest that most patients with PD may exhibit a deficient suppression of the proinflammatory response that could result in the loss of vulnerable dopaminergic neurons.

Mixed results have been published involving neutrophils and monocytes in PD. Regarding neutrophils, we reported higher levels in the whole PD cohort than in HCs. The increase in the neutrophil count was also present in the sPD and *GBA*-PD groups in our discovery cohort, but it was not replicated in the PPMI cohort. Whereas our findings are in line with other case-control studies^32–34^, others have failed to find differences in the total neutrophil count in patients with PD^35^. It has been also described that rather than quantitative changes, impairments in the neutrophil function could also be playing a role in PD^36^. However, our study design does not allow us to evidence it.

In addition, monocytes may also contribute to PD pathogenesis. In our study, even though we found a decrease in the monocyte count in the whole PD group compared to HCs, only a marginal difference was identified in the sPD group compared to HCs. Recent studies have suggested a pro-inflammatory dysregulation in monocyte subpopulations as well as an impaired phagocytic function in peripheral monocytes in PD^7,37–39^. Moreover, it has been reported that sex could influence the inflammatory activation of monocytes in patients with PD, being higher in females compared to males^40^. In this regard, we found contradicting results in both cohorts. Hence, our results should be interpreted with caution as monocyte represent a marginal proportion of the total leukocyte blood count and more specific and functional studies may be necessary to explore the role of monocytes in PD.

Furthermore, we aimed to evaluate whether there was an association between the described peripheral inflammatory immune response and the clinical trajectories of patients with PD. In our study, no correlation was found between disease duration and lymphocyte, neutrophil or monocytes counts, nor with the NLR. Even so, there is some evidence coming mainly from blood cytokines studies that suggest that the inflammatory responses is higher and play a major role in early disease and decline with time^4,7^. However, the cross-sectional design of our study does not permit us to obtain strong conclusions in this regard. Besides, a higher pro-inflammatory profile based mainly on blood cytokines and imaging features has been related to a more severe motor impairment and to the development of cognitive impairment^41–43^. Although our results in the PPMI replication cohort suggested a relationship between a higher NLR with a more severe motor impairment based on MDS-UPDRS-III scale, we failed to find an association with MOCA scores. Further studies specifically designed to assess clinical disease progression according to the peripheral immune response are needed, and also taking into account the genetic background.

It is worth mentioning that the controversial described results involving the peripheral inflammatory immune responses in patients with PD could be in part explained by the heterogeneity of populations included in the studies. To date, few studies have considered the genetic background of the individuals when studying the peripheral inflammatory immune response. The data obtained from our discovery PD cohorts’ study showed that the impaired inflammatory response observed in sPD and *GBA*-PD groups was not found in patients with *LRRK2*-PD. Furthermore, these results were replicated in the PPMI cohort as well as in sex-stratified analyses in both cohorts.

Mutations in the *LRRK2* gene are the most frequent cause of autosomal dominant PD, with clinical and pathological phenotypes very similar to sPD^44,45^. Even so, slight clinical differences have been reported between them. For example, the age at onset of *LRRK2*-PD is slightly lower than sPD and the typical male predominance seen in sPD is not seen in *LRRK2*-PD^45^. In fact, the demographics characteristics of our cohorts coincide with the previous published reports. On top of that, our results support that they could also diverge in pathophysiological mechanisms. Other groups have also attempted to explore the differences in the peripheral immune profile between *LRRK2*-PD and sPD. For example, Brockmann and collaborators found that sPD but not *LRRK2*-PD had significantly higher levels of the pro-inflammatory marker interleukin (IL)-12-p40, the anti-inflammatory marker IL-10 and the brain-derived neurotrophic factor^18^. Intriguingly, pro-inflammatory IL-12 species enhance T cell proliferation and promote inflammation by facilitating either Th1 or Th17 differentiation. Moreover, IL-10 is thought to inhibit the expansion and activation of Th1 cells and Th17 cells, and it has been suggested to modulate higher levels of the pro-inflammatory cytokine IL-12 in patients with PD^46^. In contrast, studies performed in the BEAT-PD study cohort did not find differences in the peripheral immune profile (no cellular or cytokines alterations) among patients with PD and mutations in *LRRK2* and *GBA* genes nor in non-manifesting carriers of these mutations^47,48^.

On top of that, interesting reports have also emerged from animal models. A study performed with mice overexpressing human pathogenic *LRRK2* mutations found that there was not a LRRK2 expression and/or activation in brain microglia neither a myeloid nor T cell infiltration. These findings suggested that the inflammation and neurodegeneration observed in the brain of the mutant mice could be mediated through circulating inflammatory mediators^44^. More interestingly, they found that although there was an increase in the LRRK2 expression in leukocytes, there was no difference in the expansion of the number of mononuclear cells between mutant and wild-type mice. At last, a leukocyte proteomic analysis revealed that the mutant *LRRK2* mice had an enrichment of proteins involved in immune pathways such as mitogen-activated protein kinases (MAPK) and nuclear factor kB (NF-kB) signalling. Overall, it may be hypothesised that whereas T lymphocytes could have a more prominent role in sPD etiopathogenesis, inflammation in *LRRK2*-PD could be mediated through circulating inflammatory mediators rather than through a T cell dysregulation.

Pathogenic *GBA* variants constitute the most common genetic risk factor for PD^14^. The *GBA* gene encodes the lysosomal enzyme β-glucocerebrosidase (GCase) which mediates the hydrolysis of glucocerebroside. The neurodegenerative manifestations of *GBA*-PD may arise from the loss-of-function of GCase, or from the toxic effects of accumulated lipids; however, it is becoming more apparent that inflammation plays a key role in the pathology of *GBA*-PD^7,15,49^. In both cohorts of our study, the NLR was higher in *GBA*-PD than HCs and *LRRK2*-PD, supporting the presence of a peripheral inflammatory immune response. It could be hypothesised that mechanisms underlying *GBA*-associated PD could be at least partially shared with those in sporadic PD as *GBA* acts as a risk factor and not as a causative gene in PD. For that reason, in our results, we might observe a similar peripheral inflammatory immune response compared to sPD. Moreover, the decrease in the GCase activity caused by *GBA* pathogenic variants has been shown to produce an accumulation of GCase substrates, which at the same time impairs lysosomal function and possibly leads to a decrease degradation of pathogenic synuclein, among other substrates. Some studies have suggested that when lysosome is overburdened, lysosomal vesicle contents may be released to extracellular space, activating antigen presentation, and exacerbating inflammatory immune responses through neutrophils and chronic inflammation^19,50,51^. In addition, it has been reported that monocyte GCase activity is reduced in sPD^52^. It is worth saying that *GBA*-PD patients are clinically different from *LRRK2*-PD or sPD as they commonly have a more aggressive course in terms of dementia and motor progression^12,53^. Interestingly, both dementia and severe motor impairment in PD have been related with higher levels of peripheral inflammation^43,54^. In our study, we did not found a link between cognitive impairment and peripheral inflammatory immune response, probably due to the small prevalence of cognitive impairment in our sample. Although it was beyond the scope of this study, the relationship between the peripheral inflammatory status and the more aggressive course of *GBA*-PD deserves further research. Above all, these are preliminary hypothesis which should be taken with cautious and further focused studies in *GBA*-PD should be driven to elucidate the link between *GBA* and peripheral inflammatory immune responses.

The strength of our conclusions is tempered by certain limitations. Firstly, the retrospective and cross-sectional design of this study cannot elude that some other variables out of our control might influence the results. However, we applied rigorous exclusion criteria (i.e., inflammatory diseases or cancer) to avoid confounding factors. Even though the LEDD was different among groups, previous studies that evaluated the NLR in drug-naïve PD patients suggested that this alteration was independent of LEDD^34,55^. Also due to the cross-sectional design of our study, we could not properly study whether the peripheral inflammatory immune response differs across the disease course and clinical progression of PD patients with PD. Second, other inflammatory markers were not available to support systemic inflammation. Further studies with other inflammatory markers and/or lymphocyte subpopulations might be required to further characterize the peripheral inflammatory immune response, particularly in *LRRK2*-PD patients. Despite these limitations, our strength is that the replication study performed supported our significant findings.

In conclusion, our results support the involvement of a peripheral inflammatory immune response in sPD and *GBA*-PD patients. They showed a lower lymphocyte count, a likely a higher neutrophil count and a higher NLR than HCs. However, this peripheral inflammatory immune response was not found in *LRRK2*-PD patients. These findings suggest that pathogenic inflammatory mechanisms underlying *LRRK2*-PD may be different from those underlying sPD. Further prospective and longitudinal studies are required to confirm our results. A better understanding of the different peripheral inflammatory immune response in sporadic and familial PD is likely to have clinical and therapeutic implications.

## Methods

### Participants

We included a discovery cohort consisting of 222 patients with PD and 299 HCs recruited from our Movement Disorder Clinic of the Hospital Universitario Virgen del Rocío in Seville, Spain. Patients with PD were diagnosed following the Movement Disorder Society Clinical Diagnostic Criteria^56^. Patients with PD were classified into three subgroups according to their genetic background: 132 sPD patients, 44 *LRRK2*-PD patients and 46 *GBA-*PD patients. sPD patients did not have any variants in the PD-associated genes. The *LRRK2*-PD group included 33 *LRRK2* p.G2019S PD patients and 11 *LRRK2* p.R1441G PD patients. The list of *GBA* pathogenic variants considered for the inclusion of patients in the *GBA*-PD group is shown in Table 4. HCs were volunteers from the same geographical area, and they were not considered for the study if they had any neurodegenerative disorder, a family history of PD, or a variant in *LRRK2* or *GBA* genes. In addition, exclusion criteria for all study participants were factors that could influence the immune response at the time of the blood extraction. Therefore, participants with cancer, active or chronic autoimmune, inflammatory, or infectious diseases, and those receiving immunosuppressive therapy were not considered for the study. All subjects underwent a clinical assessment at our centre and the demographic and clinical data were retrospectively obtained by consulting their medical records.

**Table 4:** List of *GBA* pathogenic variants considered for the inclusion of patients in the *GBA*-associated Parkinson’s disease group.

Allele	cDNA	Protein	Exon	Mutation severity	Discovery cohort (*n*)	PPMI replication cohort (*n*)
N370S	c.1223A/G	p.Asn409Ser	10	Mild	16	17
E326K	c.1093G > A	p.Gly365Lys	9	Mild	14	21
L444P	c.T1448C	p.Leu483Pro	11	Severe	7	3
R262C	c.901C > T	p.Arg301Cys	8	Risk	1	0
R535H	c.1604 G > A	p.Arg535His	12	Mild	5	0
F213I	c.754T > A	p.Phe252Ile	7	Severe	2	0
V457D	c.T1487A	p.Val496Asp	11	Risk	1	0
R463C	c.1504C > T	p.Arg502Cys	1	Severe	0	1
T369M	c.1223C > T	p.T369M	1	Mild	0	12

*cDNA* complementary DNA, *n* number of patients, *PPMI* Parkinson’s Progression Markers Initiative.

Total leukocyte count and subpopulations (neutrophils, lymphocytes, monocytes, eosinophils, and basophils) in peripheral blood were measured in the Central Laboratory of our center using Sysmex XN automated haematology analyser. It uses a fluorescence flow cytometry with forward-scattered, side-scattered, and side-fluorescence lights for cells determination. The NLR was calculated as absolute neutrophil count divided by absolute lymphocyte count.

For validating the peripheral immune profile in PD according to their genetic background, we used as replication cohort the Parkinson’s Progression Markers Initiative (PPMI) cohort: an international, multisite, prospective, longitudinal cohort study. PPMI began initial recruitment in 2010 enrolling untreated patients in early stages. The study expanded and reinitiated recruitment in 2020, particularly aiming to recruit patients with pathogenic genetic variants, with some differences in the eligibility criteria between the two phases, such as disease duration and medication allowed. Details regarding the PPMI study have been published^57^ and are available on the PPMI website (http://www.ppmi-info.org). The PPMI data used in this study were downloaded on June 2, 2022. We applied the same criteria as for our discovery cohort. The replication study included 401 patients with PD [281 sPD patients, 66 *LRRK2*-PD patients (54 with p.G2019S and 12 with p.R1441G mutations), 54 *GBA*-PD patients] and a group of 174 HCs. The list of *GBA* pathogenic variants considered is shown in Table 4. *GBA* mutation severity has been determined according to the literature^58–60^.

The study was approved by the local ethics committee according to the guidelines of the Helsinki declaration, and written informed consent was obtained from all study participants. The PPMI study was approved by the local institutional review boards of all participating sites.

### Genetics

Genomic DNA was isolated from peripheral blood samples by standard or automated methods (DNA Isolation Kit for Mammalian Blood, Roche, Maxwell 16 System, Promega Corporation, Madison, WI, USA; MagNA Pure LC, Roche Diagnostics, Indianapolis) in compliance with established protocols. The detailed procedures followed for the *LRRK2* and *GBA* screening are described in Supplementary Information 2.

In the PPMI replication cohort, genetic testing was done by the centralised PPMI genetic testing core. All PPMI participants subsequently had whole exome or genome sequencing. Detailed methodology is available on the PPMI website (http://www.ppmi-info.org).

### Statistical analysis

Group comparisons of categorical variables were performed using Fisher’s or the chi-squared test. Firstly, we compared the differential blood cell counts and the NLR between the total PD group and HCs using multivariate linear regression adjusting for sex and age. Secondly, the comparison was made between the different PD cohorts (sPD, *LRRK2*-PD, and *GBA*-PD) and HCs using multivariate linear regression or analysis of covariance (ANCOVA), considering age and sex as covariates. Finally, post hoc analyses with Tukey test for multiple comparisons between groups were applied. Sex-stratified analyses were also performed in both cohorts. Correlations analyses between blood cells counts and clinical characteristics were performed using Spearman or Pearson analyses, as well as multivariate linear regression, as appropriate. Saphiro–Wilk’s test was used to examine whether the different variables followed a normal distribution in the population. A *p* value < 0.05 was considered statistically significant. All statistical analyses were performed using the R version 3.5.1., and GraphPad Prism 8 software (GraphPad Software, Inc.).

### Reporting summary

Further information on research design is available in the Nature Research Reporting Summary linked to this article.

## Supplementary information

Supplementary Information

Reporting Summary

## Data Availability

The data that support the findings of this study does not present restrictions and are available upon a request that is reasonable made and that can be provided by the corresponding author [P.M. and P.G.-G.] due to participants privacy.
