# Author Correction: Peripheral inflammatory immune response differs among sporadic and familial Parkinson’s disease

**DOI:** 10.1038/s41531-023-00471-7

**Published:** 2023-02-17

**Authors:** Laura Muñoz-Delgado, Daniel Macías-García, María Teresa Periñán, Silvia Jesús, Astrid D. Adarmes-Gómez, Marta Bonilla Toribio, Dolores Buiza Rueda, María del Valle Jiménez-Jaraba, Belén Benítez Zamora, Rafael Díaz Belloso, Sergio García-Díaz, Miguel Martín-Bórnez, Rocío Pineda Sánchez, Fátima Carrillo, Pilar Gómez-Garre, Pablo Mir

**Affiliations:** 1 Unidad de Trastornos del Movimiento, Servicio de Neurología y Neurofisiología Clínica, Instituto de Biomedicina de Sevilla, Hospital Universitario Virgen del Rocío/CSIC/Universidad de Sevilla, Seville, Spain; 2 Centro de Investigación Biomédica en Red sobre Enfermedades Neurodegenerativas (CIBERNED), Madrid, Spain; 3 Departamento de Medicina, Facultad de Medicina, Universidad de Sevilla, Seville, Spain

**Keywords:** Parkinson's disease, Inflammation

Correction to: *npj Parkinson’s Disease* 10.1038/s41531-023-00457-5, published online 31 January 2023

In this article the affiliation details for Author Pablo Mir were incorrectly given as ‘Departamento de Medicina, Universidad de Sevilla, Seville, Spain’ but should have been ‘Departamento de Medicina, Facultad de Medicina, Universidad de Sevilla, Seville, Spain’. The original article has been corrected.

