# Supplementary material for: Peripheral inflammatory immune response differs among sporadic and familial Parkinson’s disease
Source: NPJ Parkinsons Dis. 2023 Jan 31;9:12. doi: 10.1038/s41531-023-00457-5 (PMC9889312; doi:10.1038/s41531-023-00457-5)
Supplement: Supplementary file 1 — Supplementary Information [file 41531_2023_457_MOESM1_ESM.pdf]

Supplementary Information 1. Results

Part A. Post Hoc Comparisons to determine the differences in the peripheral immune response between groups.

Supplementary Table 1. Discovery cohort. Post Hoc Comparisons – Lymphocyte count

Group A	Group B	Mean difference (Group A-B)	95% CI for Mean Difference		SE	t	p tukey
			Lower	Upper			
HCs	*GBA*-PD	0.273	0.019	0.526	0.098	2.771	0.029*
	sPD	0.327	0.158	0.497	0.066	4.972	< 0.001*
	LRRK2-PD	0.099	-0.159	0.358	0.100	0.990	0.755
GBA-PD	sPD	0.054	-0.223	0.331	0.107	0.506	0.958
	LRRK2-PD	-0.173	-0.513	0.166	0.132	-1.317	0.553
sPD	*LRRK2*-PD	-0.228	-0.508	0.052	0.109	-2.096	0.156

Note: P-value and confidence intervals adjusted for comparing a family of 4 estimates (confidence intervals corrected using the tukey method). * A p value < 0.05 was considered statistically significant.

Abbreviations: SE, standard error; HCs, healthy controls; sPD, sporadic Parkinson's disease; *GBA*-PD, patients with *GBA*-associated Parkinson's disease; *LRRK2*-PD, patients with *LRRK2*-associated Parkinson's disease.

Supplementary Table 2. PPMI replication cohort. Post Hoc Comparisons – Lymphocyte count

Group A	Group B	Mean difference (Group A-B)	95% CI for Mean Difference		SE	t	p tukey
			Lower	Upper			
GBA-PD	HCs	-0.195	-0.460	0.069	0.103	-1.901	0.229
	sPD	-0.012	-0.265	0.241	0.098	-0.120	0.999
	LRRK2-PD	-0.204	-0.516	0.108	0.121	-1.682	0.334
HCs	sPD	0.184	0.019	0.348	0.064	2.880	0.021*
	LRRK2-PD	-0.008	-0.255	0.239	0.096	-0.086	1.000
sPD	*LRRK2*-PD	-0.192	-0.426	0.043	0.091	-2.108	0.152

Note: P-value and confidence intervals adjusted for comparing a family of 4 estimates (confidence intervals corrected using the tukey method). *A p value < 0.05 was considered statistically significant.

Abbreviations: SE, standard error; HCs, healthy controls; sPD, sporadic Parkinson's disease; *GBA*-PD, patients with *GBA*-associated Parkinson's disease; *LRRK2*-PD, patients with *LRRK2*-associated Parkinson's disease.

Supplementary Table 3. Discovery cohort. Post Hoc Comparisons – Neutrophil count

Group A	Group B	Mean difference (Group A-B)	95% CI for Mean Difference		SE	t	p tukey
			Lower	Upper			
HCs	*GBA*-PD	-0.410	-0.924	0.103	0.199	-2.059	0.168
	sPD	-0.287	-0.630	0.056	0.133	-2.155	0.137
	*LRRK*2-PD	-0.141	-0.665	0.383	0.203	-0.694	0.899
GBA-PD	sPD	0.123	-0.437	0.684	0.217	0.567	0.942
	LRRK2-PD	0.269	-0.417	0.956	0.266	1.010	0.744
sPD	*LRRK2*-PD	0.146	-0.421	0.713	0.220	0.663	0.911

Note: P-value and confidence intervals adjusted for comparing a family of 4 estimates (confidence intervals corrected using the tukey method). A p value < 0.05 was considered statistically significant.

Abbreviations: SE, standard error; HCs, healthy controls; sPD, sporadic Parkinson's disease; *GBA*-PD, patients with *GBA*-associated Parkinson's disease; *LRRK2*-PD, patients with *LRRK2*-associated Parkinson's disease.

Supplementary Table 4. PPMI replication cohort. Post Hoc Comparisons – Neutrophil count

Group A	Group B	Mean difference (Group A-B)	95% CI for Mean Difference		SE	t	p tukey
			Lower	Upper			
GBA-PD	HCs	0.573	0.077	1.069	0.193	2.976	0.016*
	sPD	0.332	-0.142	0.806	0.184	1.803	0.273
	LRRK2-PD	0.265	-0.320	0.849	0.227	1.167	0.648
HCs	sPD	-0.241	-0.549	0.067	0.119	-2.018	0.182
	LRRK2-PD	-0.308	-0.772	0.155	0.180	-1.714	0.317
sPD	*LRRK2*-PD	-0.067	-0.507	0.372	0.171	-0.394	0.979

Note: P-value and confidence intervals adjusted for comparing a family of 4 estimates (confidence intervals corrected using the tukey method). *A p value < 0.05 was considered statistically significant.

Abbreviations: SE, standard error; HCs, healthy controls; sPD, sporadic Parkinson's disease; *GBA*-PD, patients with *GBA*-associated Parkinson's disease; *LRRK2*-PD, patients with *LRRK2*-associated Parkinson's disease.

Supplementary Table 5. Discovery cohort. Post Hoc Comparisons – Monocyte count

Group A	Group B	Mean difference (Group A-B)	95% CI for Mean Difference		SE	t	p tukey
			Lower	Upper			
HCs	*GBA*-PD	0.037	-0.081	0.156	0.046	0.814	0.848
	sPD	0.079	-5.309e-5	0.158	0.031	2.576	0.050
	LRRK2-PD	0.021	-0.100	0.142	0.047	0.445	0.971
GBA-PD	sPD	0.042	-0.088	0.171	0.050	0.832	0.839
	LRRK2-PD	-0.017	-0.175	0.142	0.061	-0.270	0.993
sPD	*LRRK2*-PD	-0.058	-0.189	0.073	0.051	-1.149	0.659

Note: P-value and confidence intervals adjusted for comparing a family of 4 estimates (confidence intervals corrected using the tukey method). A p value < 0.05 was considered statistically significant.
Abbreviations: SE, standard error; HCs, healthy controls; sPD, sporadic Parkinson's disease; *GBA*-PD, patients with *GBA*-associated Parkinson's disease; *LRRK2*-PD, patients with *LRRK2*-associated Parkinson's disease.

Supplementary Table 6. PPMI replication cohort. Post Hoc Comparisons – Monocyte count

Group A	Group B	Mean difference (Group A-B)	95% CI for Mean Difference		SE	t	p tukey
			Lower	Upper			
GBA-PD	HCs	-0.002	-0.055	0.051	0.021	-0.076	1.000
	sPD	0.030	-0.020	0.081	0.020	1.542	0.413
	LRRK2-PD	0.032	-0.030	0.094	0.024	1.319	0.551
HCs	sPD	0.032	-0.001	0.065	0.013	2.498	0.061
	LRRK2-PD	0.034	-0.016	0.083	0.019	1.746	0.301
sPD	*LRRK2*-PD	0.002	-0.045	0.049	0.018	0.091	1.000

Note: P-value and confidence intervals adjusted for comparing a family of 4 estimates (confidence intervals corrected using the tukey method). *A p value < 0.05 was considered statistically significant.
Abbreviations: SE, standard error; HCs, healthy controls; sPD, sporadic Parkinson's disease; *GBA*-PD, patients with *GBA*-associated Parkinson's disease; *LRRK2*-PD, patients with *LRRK2*-associated Parkinson's disease.

Supplementary Table 7. Discovery cohort. Post Hoc Comparisons – Neutrophil-to-lymphocyte ratio

Group A	Group B	Mean difference (Group A-B)	95% CI for Mean Difference		SE	t	p tukey
			Lower	Upper			
HCs	*GBA*-PD	-0.599	-1.000	-0.197	0.156	-3.840	< .001*
	sPD	-0.590	-0.859	-0.322	0.104	-5.664	< .001*
	LRRK2-PD	-0.120	-0.530	0.290	0.159	-0.753	0.875
GBA-PD	sPD	0.008	-0.430	0.447	0.170	0.050	1.000
	LRRK2-PD	0.479	-0.059	1.016	0.208	2.297	0.100
sPD	*LRRK2*-PD	0.470	0.027	0.914	0.172	2.733	0.033*

Note: P-value and confidence intervals adjusted for comparing a family of 4 estimates (confidence intervals corrected using the tukey method). *A p value < 0.05 was considered statistically significant.

Abbreviations: SE, standard error; HCs, healthy controls; sPD, sporadic Parkinson's disease; *GBA*-PD, patients with *GBA*-associated Parkinson's disease; *LRRK2*-PD, patients with *LRRK2*-associated Parkinson's disease.

Supplementary Table 8. PPMI replication cohort. Post Hoc Comparisons – Neutrophil-to-lymphocyte ratio

Group A	Group B	Mean difference (Group A-B)	95% CI for Mean Difference		SE	t	p tukey
			Lower	Upper			
GBA-PD	HCs	0.532	0.128	0.935	0.156	3.397	0.004*
	sPD	0.177	-0.209	0.563	0.150	1.181	0.639
	LRRK2-PD	0.411	-0.064	0.886	0.184	2.228	0.117
HCs	sPD	-0.355	-0.605	-0.104	0.097	-3.643	0.002*
	LRRK2-PD	-0.121	-0.497	0.256	0.146	-0.826	0.842
sPD	*LRRK2*-PD	0.234	-0.124	0.592	0.139	1.685	0.332

Note: P-value and confidence intervals adjusted for comparing a family of 4 estimates (confidence intervals corrected using the tukey method). *A p value < 0.05 was considered statistically significant.

Abbreviations: SE, standard error; HCs, healthy controls; sPD, sporadic Parkinson's disease; *GBA*-PD, patients with *GBA*-associated Parkinson's disease; *LRRK2*-PD, patients with *LRRK2*-associated Parkinson's disease.

Part B. Sex-stratified analyses to determine the differences in the peripheral immune response between groups.

Supplementary Table 9. Demographic and clinical data, and peripheral immune profile of healthy controls and Parkinson's disease genetic cohorts of the discovery cohort, sex-stratified

	Sex	HCs	sPD	*LRRK2*-PD	*GBA*-PD	*F value*	*P value*
**N**	M	153	80	17	27		
	F	146	52	27	19		
**Age (y), mean ± SD**	M	60.09 ± 15.74	63.95 ± 11.95	64.35 ± 11.35	53.63 ± 9.96	4.20	**< 0.05**[1]
	F	59.80 ± 14.51	66.81 ± 11.31	61.56 ± 11.25	60.90 ± 8.46	3.64	**0.01**[1]
**Age of onset (y), mean ± SD**	M	-	56.25 ± 12.67	55.24 ± 13.71	46.82 ± 10.19	6.01	**< 0.05**[1]
	F		58.21 ± 11.25	52.44 ± 11.22	51.26 ± 8.47	4.18	**0.02**[1]
**Disease duration (y), mean ± SD**	M		7.70 ± 5.84	9.12 ± 7.05	6.82 ± 6.10	0.75	0.47[1]
	F		8.60 ± 5.06	9.11 ± 5.96	9.63 ± 5.05	0.28	0.75[1]
**HY in OFF state, mean ± SD**	M		2.42 ± 0.87	2.56 ± 0.63	2.26 ± 0.86	0.70	0.50[1]
	F		2.78 ± 1.11	2.82 ± 0.92	3 ± 0.91	0.31	0.73[1]
**LEDD, mean ± SD**	M		740.51 ± 471.61	1085.53 ± 593.88	810.64 ± 714.70	2.77	0.07[1]
	F		780.30 ± 523.89	943.92 ± 531.60	948.32 ± 486.84	1.23	0.30[1]
**Leukocyte count, mean ± SD**	M	7 ± 1.58	7.02 ± 1.35	7.24 ± 1.50	6.93 ± 1.63	1.56	0.18[2]
	F	6.77 ± 1.82	6.39 ± 1.67	6.54 ± 1.48	6.91 ± 1.41	0.70	0.59[2]
**Lymphocyte count, mean ± SD**	M	2.17 ± 0.64	1.84 ± 0.63	2.11 ± 0.66	1.87 ± 0.58	4.06	**0.003**[2]
	F	2.13 ± 0.66	1.72 ± 0.61	1.97 ± 0.40	1.95 ± 0.57	5.69	**< 0.001**[2]
**Neutrophil count, mean ± SD**	M	3.99 ± 1.16	4.41 ± 1.16	4.29 ± 1.03	4.35 ± 1.42	3.97	**0.004**[2]
	F	3.86 ± 1.33	4.04 ± 1.40	3.91 ± 1.25	4.29 ± 1.36	0.54	0.71[2]
**Monocyte count, mean ± SD**	M	0.53 ± 0.17	0.48 ± 0.13	0.58 ± 0.21	0.51 ± 0.14	3.31	**0.011**[2]
	F	0.50 ± 0.49	0.39 ± 0.11	0.43 ± 0.15	0.43 ± 0.13	0.92	0.46[2]
**Eosinophil count, mean ± SD**	M	0.25 ± 0.39	0.18 ± 0.13	0.20 ± 0.14	0.15 ± 0.12	1.69	0.15[2]
	F	0.23 ± 0.34	0.23 ± 0.82	0.14 ± 0.08	0.16 ± 0.11	0.18	0.86[2]
**Basophil count, mean ± SD**	M	0.12 ± 0.76	0.02 ± 0.03	0.04 ± 0.04	0.03 ± 0.02	0.47	0.76[2]
	F	0.03 ± 0.03	0.02 ± 0.03	0.03 ± 0.03	0.03 ± 0.03	1.68	0.16[2]
**NLR, mean ± SD**	M	2.01 ± 0.94	2.66 ± 1.10	2.21 ± 0.79	2.58 ± 1.33	6.77	**<0.001**[2]
	F	1.94 ± 0.83	2.58 ± 1.18	2.05 ± 0.75	2.50 ± 1.36	5.60	**<0.001**[2]

Abbreviations: HC, healthy control; sPD, sporadic Parkinson's disease; *GBA*-PD, patients with *GBA*-associated Parkinson's disease; *LRRK2*-PD, patients with *LRRK2*-associated Parkinson's disease; N, total number of individuals; M, male; F, female; y, years; SD, standard deviation; HY, Hoehn & Yahr stage; LEDD, levodopa equivalent daily dose; NLR, neutrophil-to-lymphocyte ratio. Note: All blood cell counts are expressed as x10^3 cells/µL. [1]Based on linear regression and analysis of covariance (ANCOVA); [2]Based on multivariate linear regression, adjusting for age. *A p value < 0.05 was considered statistically significant and bolded.

Supplementary Table 10. Discovery cohort - Males. Post Hoc Comparisons – Lymphocyte count

Group A	Group B	Mean difference (Group A-B)	95% CI for Mean Difference		SE	t	p tukey
			Lower	Upper			
HCs	*GBA*-PD	0.333	-0.011	0.676	0.133	2.502	0.062
	sPD	0.307	0.081	0.534	0.088	3.502	0.003*
	LRRK2-PD	0.037	-0.381	0.456	0.162	0.230	0.996
GBA-PD	sPD	-0.025	-0.396	0.345	0.143	-0.177	0.998
	LRRK2-PD	-0.295	-0.806	0.216	0.198	-1.495	0.442
sPD	*LRRK2*-PD	-0.270	-0.706	0.166	0.169	-1.602	0.379

Note: P-value and confidence intervals adjusted for comparing a family of 4 estimates (confidence intervals corrected using the tukey method). *A p value < 0.05 was considered statistically significant.

Abbreviations: SE, standard error; HCs, healthy controls; sPD, sporadic Parkinson's disease; *GBA*-PD, patients with *GBA*-associated Parkinson's disease; *LRRK2*-PD, patients with *LRRK2*-associated Parkinson's disease.

Supplementary Table 11. Discovery cohort - Females. Post Hoc Comparisons – Lymphocyte count

Group A	Group B	Mean difference (Group A-B)	95% CI for Mean Difference		SE	t	p tukey
			Lower	Upper			
HCs	*GBA*-PD	0.171	-0.213	0.555	0.148	1.153	0.657
	sPD	0.349	0.089	0.609	0.100	3.473	0.003*
	LRRK2-PD	0.141	-0.189	0.471	0.128	1.102	0.689
GBA-PD	sPD	0.178	-0.247	0.602	0.164	1.084	0.700
	LRRK2-PD	-0.031	-0.502	0.441	0.182	-0.168	0.998
sPD	*LRRK2*-PD	-0.208	-0.584	0.167	0.145	1.435	0.479

Note: P-value and confidence intervals adjusted for comparing a family of 4 estimates (confidence intervals corrected using the tukey method). *A p value < 0.05 was considered statistically significant.

Abbreviations: SE, standard error; HCs, healthy controls; sPD, sporadic Parkinson's disease; *GBA*-PD, patients with *GBA*-associated Parkinson's disease; *LRRK2*-PD, patients with *LRRK2*-associated Parkinson's disease.

Supplementary Table 12. Discovery cohort - Males. Post Hoc Comparisons – Neutrophil count

Group A	Group B	Mean difference (Group A-B)	95% CI for Mean Difference		SE	t	p tukey
			Lower	Upper			
HCs	*GBA*-PD	-0.438	-1.076	0.200	0.247	-1.776	0.287
	sPD	-0.383	-0.804	0.038	0.163	-2.354	0.089
	LRRK2-PD	-0.255	-1.032	0.521	0.300	-0.850	0.831
GBA-PD	sPD	0.055	-0.633	0.743	0.266	0.206	0.997
	LRRK2-PD	0.183	-0.765	1.131	0.367	0.499	0.959
sPD	*LRRK2*-PD	0.128	-0.681	0.937	0.313	0.410	0.977

Note: P-value and confidence intervals adjusted for comparing a family of 4 estimates (confidence intervals corrected using the tukey method). *A p value < 0.05 was considered statistically significant.

Abbreviations: SE, standard error; HCs, healthy controls; sPD, sporadic Parkinson's disease; *GBA*-PD, patients with *GBA*-associated Parkinson's disease; *LRRK2*-PD, patients with *LRRK2*-associated Parkinson's disease.

Supplementary Table 13. Discovery cohort - Males. Post Hoc Comparisons – Monocyte count

Group A	Group B	Mean difference (Group A-B)	95% CI for Mean Difference		SE	t	p tukey
			Lower	Upper			
HCs	*GBA*-PD	0.010	-0.076	0.097	0.034	0.307	0.997
	sPD	0.051	-0.006	0.108	0.022	2.321	0.063
	LRRK2-PD	-0.046	-0.151	0.059	0.041	-1.128	0.673
GBA-PD	sPD	0.041	-0.052	0.134	0.036	1.137	0.530
	LRRK2-PD	-0.056	-0.185	0.072	0.050	-1.130	0.714
sPD	*LRRK2*-PD	-0.097	-0.207	0.013	0.042	-2.290	0.085

Note: P-value and confidence intervals adjusted for comparing a family of 4 estimates (confidence intervals corrected using the tukey method). *A p value < 0.05 was considered statistically significant.

Abbreviations: SE, standard error; HCs, healthy controls; sPD, sporadic Parkinson's disease; *GBA*-PD, patients with *GBA*-associated Parkinson's disease; *LRRK2*-PD, patients with *LRRK2*-associated Parkinson's disease.

Supplementary Table 14. Discovery cohort – Males. Post Hoc Comparisons – Neutrophil-to-lymphocyte ratio

Group A	Group B	Mean difference (Group A-B)	95% CI for Mean Difference		SE	t	p tukey
			Lower	Upper			
HCs	*GBA*-PD	-0.650	-1.202	-0.098	0.214	-3.042	0.014
	sPD	-0.612	-0.976	-0.247	0.141	-4.338	<.001
	LRRK2-PD	-0.154	-0.827	0.518	0.260	-0.594	0.934
GBA-PD	sPD	0.038	-0.557	0.634	0.230	0.166	0.998
	LRRK2-PD	0.496	-0.326	1.317	0.318	1.560	0.403
sPD	*LRRK2*-PD	0.457	-0.243	1.158	0.271	1.688	0.332

Note: P-value and confidence intervals adjusted for comparing a family of 4 estimates (confidence intervals corrected using the tukey method). *A p value < 0.05 was considered statistically significant.

Abbreviations: SE, standard error; HCs, healthy controls; sPD, sporadic Parkinson's disease; *GBA*-PD, patients with *GBA*-associated Parkinson's disease; *LRRK2*-PD, patients with *LRRK2*-associated Parkinson's disease.

Supplementary Table 15. Discovery cohort - Females. Post Hoc Comparisons – Neutrophil-to-lymphocyte ratio

Group A	Group B	Mean difference (Group A-B)	95% CI for Mean Difference		SE	t	p tukey
			Lower	Upper			
HCs	*GBA*-PD	-0.541	-1.140	0.058	0.232	-2.335	0.093
	sPD	-0.565	-0.971	-0.160	0.157	-3.605	0.002
	LRRK2-PD	-0.091	-0.606	0.424	0.199	-0.459	0.968
GBA-PD	sPD	-0.024	-0.687	0.638	0.256	-0.095	1.000
	LRRK2-PD	0.450	-0.286	1.185	0.284	1.581	0.392
sPD	*LRRK2*-PD	0.474	-0.112	1.060	0.227	2.092	0.159

Note: P-value and confidence intervals adjusted for comparing a family of 4 estimates (confidence intervals corrected using the tukey method). *A p value < 0.05 was considered statistically significant.

Abbreviations: SE, standard error; HCs, healthy controls; sPD, sporadic Parkinson's disease; *GBA*-PD, patients with *GBA*-associated Parkinson's disease; *LRRK2*-PD, patients with *LRRK2*-associated Parkinson's disease.

Supplementary Table 16. Demographic and clinical data, and peripheral immune profile of healthy controls and Parkinson's disease genetic cohorts of the PPMI cohort, sex-stratified

	Sex	HCs	sPD	*LRRK2*-PD	*GBA*-PD	*F value*	*P value*
**N**	M	111	194	31	30		
	F	62	87	35	24		
**Age (y), mean ± SD**	M	61.38 ± 11.34	61.89 ± 9.72	60.52 ± 10.12	59.9 ± 8.51	0.44	0.73[1]
	F	56.98 ± 13.34	60.53 ± 9.17	62.89 ± 10.29	58.71 ±11.57	2.46	0.06[1]
**Age of onset (y), mean ± SD**	M	-	59.96 ± 10	55.76 ± 10.77	57.61 ± 9.19	2.77	0.06[1]
	F	-	58.59 ± 9.37	57.98 ± 10.44	56.17 ± 11.93	0.54	0.58[1]
**Disease duration (y), mean ± SD**	M	-	1.92 ± 2.03	4.75 ± 3.13	2.3 ± 2.08	22.18	**< 0.001**[1]
	F	-	1.94 ± 1.33	4.53 ± 2.43	2.54 ± 2.27	25.07	**< 0.001**[1]
**HY in OFF state, mean ± SD**	M	-	1.58 ± 0.50	1.89 ± 0.64	1.64 ± 0.49	4.28	**< 0.05**[1]
	F	-	1.55 ± 0.50	2 ± 0.61	1.61 ± 0.58	7.46	**< 0.001**[1]
**MDS-UPDRS-III in OFF state, mean ± SD**	M	-	21.14 ± 8.85	20.54 ± 8.91	25.18 ± 12.21	2.50	0.08[1]
	F	-	20.69 ± 8.84	17.82 ± 7.39	21.61 ± 9.37	1.50	0.23[1]
**LEDD, mean ± SD**	M	-	2.37 ± 24.36	411.41 ± 362.43	62.98 ± 168.01	115.07	**< 0.001**[1]
	F	-	0	443.05 ± 322.75	85.42 ± 213.40	76.87	**< 0.001**[1]
**Leukocyte count, mean ± SD**	M	6.17 ± 1.88	6.34 ±1.52	6.50 ±2.19	6.55 ± 1.38	0.59	0.62[2]
	F	6.14 ± 1.68	5.82 ±1.37	6.14 ± 1.30	6.39 ± 1.57	1.29	0.28[2]
**Lymphocyte count, mean ± SD**	M	1.80 ± 1.03	1.67 ± 0.53	1.78 ± 0.63	1.59 ± 0.46	1.15	0.33[2]
	F	1.93 ± 0.65	1.63 ± 0.45	1.93 ± 0.59	1.74 ± 0.44	4.83	**0.0009**[2]
**Neutrophil count, mean ± SD**	M	3.70 ± 1.14	4.05 ± 1.26	4.20 ± 1.72	4.30 ± 1.20	2.19	0.07[2]
	F	3.62 ± 1.25	3.67 ± 1.15	3.71 ± 1.03	4.12 ± 1.32	1.09	0.34[2]
**Monocyte count, mean ± SD**	M	0.43 ± 0.17	0.41 ± 0.12	0.39 ± 0.13	0.44 ± 0.13	1.26	0.29[2]
	F	0.37 ± 0.12	0.33 ± 0.12	0.35 ± 0.10	0.36 ± 0.09	2.21	0.07[2]
**Eosinophil count, mean ± SD**	M	0.18 ± 0.13	0.17 ± 0.12	0.15 ± 0.12	0.16 ± 0.10	0.62	0.60[2]
	F	0.16 ± 0.12	0.15 ± 0.18	0.16 ± 0.09	0.13 ± 0.07	0.54	0.80[2]
**Basophil count, mean ± SD**	M	0.05 ± 0.03	0.05 ± 0.03	0.04 ± 0.03	0.05 ± 0.04	0.62	0.60[2]
	F	0.05 ± 0.03	0.04 ± 0.03	0.04 ± 0.02	0.04 ± 0.02	0.34	0.80[2]
**NLR, mean ± SD**	M	2.27 ± 0.79	2.62 ± 1.06	2.58 ± 1.13	2.88 ± 1.1	3.87	**0.004**[2]
	F	2.02 ± 0.76	2.44 ±1.39	2.01 ± 0.60	2.44 ± 0.81	4.15	**0.003**[2]

Abbreviations: HC, healthy control; PD, Parkinson's disease; N, total number of individuals; M, male; F, female; y, years; SD, standard deviation; HY, Hoehn & Yahr stage; MDS-UPDRS-III, MDS Unified Parkinson's Disease Rating Scale part III scale; LEDD, levodopa equivalent daily dose; NLR, neutrophil-to-lymphocyte ratio. Note: All blood cell counts were expressed as x10^3 cells/µL. [1]Based on linear regression and analysis of covariance (ANCOVA); [2]Based on multivariate linear regression, adjusting for age. *A p value < 0.05 was considered statistically significant and bolded.

Supplementary Table 17. PPMI replication cohort - Females. Post Hoc Comparisons – Lymphocyte count

Group A	Group B	Mean difference (Group A-B)	95% CI for Mean Difference		SE	t	p tukey
			Lower	Upper			
GBA-PD	HCs	-0.179	-0.512	0.154	0.129	-1.390	0.507
	sPD	0.095	-0.224	0.415	0.123	0.774	0.866
	LRRK2-PD	-0.220	-0.589	0.148	0.142	-1.548	0.411
HCs	sPD	0.274	0.042	0.506	0.090	3.059	0.013
	LRRK2-PD	-0.042	-0.339	0.256	0.115	-0.362	0.984
sPD	*LRRK2*-PD	-0.316	-0.594	-0.038	0.107	-2.944	0.019

Note: P-value and confidence intervals adjusted for comparing a family of 4 estimates (confidence intervals corrected using the tukey method). *A p value < 0.05 was considered statistically significant.
Abbreviations: SE, standard error; HCs, healthy controls; sPD, sporadic Parkinson's disease; *GBA*-PD, patients with *GBA*-associated Parkinson's disease; *LRRK2*-PD, patients with *LRRK2*-associated Parkinson's disease.

Supplementary Table 18. PPMI replication cohort - Males. Post Hoc Comparisons – Neutrophil count

Group A	Group B	Mean difference (Group A-B)	95% CI for Mean Difference		SE	t	p tukey
			Lower	Upper			
GBA-PD	HCs	0.596	-0.076	1.269	0.261	2.290	0.102
	sPD	0.249	-0.392	0.891	0.249	1.003	0.748
	LRRK2-PD	0.105	-0.732	0.941	0.324	0.323	0.988
HCs	sPD	-0.347	-0.736	0.041	0.151	-2.306	0.098
	LRRK2-PD	-0.492	-1.155	0.172	0.257	-1.913	0.224
sPD	*LRRK2*-PD	-0.144	-0.776	0.488	0.245	-0.590	0.935

Note: P-value and confidence intervals adjusted for comparing a family of 4 estimates (confidence intervals corrected using the tukey method). *A p value < 0.05 was considered statistically significant.
Abbreviations: SE, standard error; HCs, healthy controls; sPD, sporadic Parkinson's disease; *GBA*-PD, patients with *GBA*-associated Parkinson's disease; *LRRK2*-PD, patients with *LRRK2*-associated Parkinson's disease.

Supplementary Table 19. PPMI replication cohort - Females. Post Hoc Comparisons – Monocyte count

Group A	Group B	Mean difference (Group A-B)	95% CI for Mean Difference		SE	t	p tukey
			Lower	Upper			
GBA-PD	HCs	-0.015	-0.086	0.056	0.027	-0.545	0.948
	sPD	0.034	-0.034	0.102	0.026	1.295	0.567
	LRRK2-PD	0.019	-0.060	0.097	0.030	0.626	0.924
HCs	sPD	0.049	-0.005	0.098	0.019	2.564	0.054
	LRRK2-PD	0.034	-0.029	0.097	0.024	1.387	0.509
sPD	*LRRK2*-PD	-0.015	-0.074	0.044	0.023	-0.659	0.912

Note: P-value and confidence intervals adjusted for comparing a family of 4 estimates (confidence intervals corrected using the tukey method). *A p value < 0.05 was considered statistically significant.

Abbreviations: SE, standard error; HCs, healthy controls; sPD, sporadic Parkinson's disease; *GBA*-PD, patients with *GBA*-associated Parkinson's disease; *LRRK2*-PD, patients with *LRRK2*-associated Parkinson's disease.

Supplementary Table 20. PPMI replication cohort - Males. Post Hoc Comparisons – Neutrophil-to-lymphocyte ratio

Group A	Group B	Mean difference (Group A-B)	95% CI for Mean Difference		SE	t	p tukey
			Lower	Upper			
GBA-PD	HCs	0.627	0.102	1.151	0.203	3.085	0.012
	sPD	0.281	-0.219	0.781	0.194	1.451	0.469
	LRRK2-PD	0.305	-0.347	0.957	0.253	1.206	0.624
HCs	sPD	-0.346	-0.649	-0.042	0.117	-2.943	0.018*
	LRRK2-PD	-0.322	-0.839	0.195	0.200	-1.607	0.376
sPD	*LRRK2*-PD	0.024	-0.469	0.516	0.191	0.123	0.999

Note: P-value and confidence intervals adjusted for comparing a family of 4 estimates (confidence intervals corrected using the tukey method). *A p value < 0.05 was considered statistically significant.

Abbreviations: SE, standard error; HCs, healthy controls; sPD, sporadic Parkinson's disease; *GBA*-PD, patients with *GBA*-associated Parkinson's disease; *LRRK2*-PD, patients with *LRRK2*-associated Parkinson's disease.

Supplementary Table 21. PPMI replication cohort - Females. Post Hoc Comparisons – Neutrophil-to-lymphocyte ratio

Group A	Group B	Mean difference (Group A-B)	95% CI for Mean Difference		SE	t	p tukey
			Lower	Upper			
GBA-PD	HCs	0.394	-0.255	1.042	0.250	1.573	0.396
	sPD	0.040	-0.582	0.662	0.240	0.167	0.998
	LRRK2-PD	0.503	-0.214	1.221	0.277	1.817	0.268
HCs	sPD	-0.353	-0.805	0.098	0.174	-2.027	0.181
	LRRK2-PD	0.110	-0.469	0.688	0.223	0.491	0.961
sPD	*LRRK2*-PD	0.463	-0.078	1.004	0.209	2.219	0.122

Note: P-value and confidence intervals adjusted for comparing a family of 4 estimates (confidence intervals corrected using the tukey method). *A p value < 0.05 was considered statistically significant.

Abbreviations: SE, standard error; HCs, healthy controls; sPD, sporadic Parkinson's disease; *GBA*-PD, patients with *GBA*-associated Parkinson's disease; *LRRK2*-PD, patients with *LRRK2*-associated Parkinson's disease.

Part C. Peripheral inflammatory immune response according to clinical characteristics of patients with PD.

a. Disease duration

To evaluate whether there was a correlation between the peripheral inflammatory immune response and the disease duration, we performed Spearman's correlation analyses in both cohorts and in the different genetic subgroups. Detailed results can be found in Supplementary table 22. When Spearman's correlations were statistically significant (p value < 0.05), we further performed a multivariate linear regression analysis adjusting for age, gender, and LEDD to control for possible confounding factors.

Firstly, in our discovery cohort, we found a statistically significant correlation between the disease duration and lymphocytes and the NLR in the whole PD cohort. However, these results were not replicated in the PPMI cohort. Moreover, these correlations were not found in the multivariate linear analyses adjusting for age, sex and LEDD. Multivariate linear regression analysis [F (4,216) = 22.07, p<0.001] showed that the lymphocyte count was not statistically significant (p=0.80), whereas age was a clear confounding factor (p<0.05). Likewise, multivariate linear regression analysis [F (4,216) = 22.05, p<0.001] showed that the NLR was not statistically significant (p=0.80), whereas age was a clear confounding factor (p<0.05).

When evaluating Parkinson's disease genetic subgroups, a correlation between disease duration and the NLR could be observed in the *LRRK2*-PD subgroup in the Spearman's correlation. However, this correlation was not statistically significant after performing a multivariate linear regression analysis in both cohorts [F(4,39)=1.83, p=0.14 in the discovery cohort, and F(4,63)=2.21, p=0.07 in the PPMI cohort].

In conclusion, no correlation was found between disease duration and the peripheral immune response studied in either the discovery cohort or the PPMI replication cohort.

Supplementary Table 22. The correlation (Spearman) between disease duration and the different blood cells counts as well as the NLR in patients with Parkinson's disease from the discovery and the PPMI replication cohorts

	Disease duration (y)							
	Whole PD cohort		sPD		*LRRK2*-PD		*GBA*-PD	
	Discovery (n=222)	PPMI (n=401)	Discovery (n=132)	PPMI (n=281)	Discovery (n=44)	PPMI (n=66)	Discovery (n=46)	PPMI (n=54)
**Lymphocytes (x10^3 cells/µL)**	- 0.134 (0.05)	+ 0.03 (0.54)	- 0.13 (0.14)	- 0.02 (0.70)	- 0.21 (0.17)	+ 0.07 (0.58)	- 0.08 (0.60)	-0.11 (0.42)
**Neutrophils (x10^3 cells/µL)**	+ 0.06 (0.40)	+ 0.03 (0.57)	+ 0.08 (0.38)	- 0.04 (0.55)	+ 0.17 (0.27)	+ 0.34 (0.006)*	- 0.08 (0.62)	+0.11 (0.42)
**Monocytes (x10^3 cells/µL)**	+ 0.05 (0.51)	- 0.07 (0.19)	+ 0.03 (0.74)	- 0.10 (0.09)	+ 0.03 (0.83)	+ 0.04 (0.73)	+ 0.05 (0.77)	+0.17 (0.23)
**NLR**	+ 0.14 (0.04)*	+ 0.01 (0.80)	+ 0.15 (0.09)	- 0.01 (0.82)	+ 0.33 (0.03)*	+ 0.28 (0.02)*	- 0.03 (0.82)	+0.21 (0.14)

Note: values expressed as: rho correlation coefficient (p value). *A p value < 0.05 was considered statistically significant. Abbreviations: y, years; sPD, sporadic Parkinson's disease; *GBA*-PD, patients with *GBA*-associated Parkinson's disease; *LRRK2*-PD, patients with *LRRK2*-associated Parkinson's disease; n, number of subjects; NLR, neutrophil-to-lymphocyte ratio.

b. Motor impairment

Motor impairment in patients with PD was assessed based on HY stage and MDS-UPDRS-III scale, both in OFF state. Data concerning Hoehn & Yahr stage was available in both cohorts. Unfortunately, UPDRS part III scale was not available in the discovery cohort as data was obtained retrospectively and UPDRS scale was not previously recorded in the clinics.

Concerning HY stage, data are shown in Supplementary Table 23 below. Remarkably, a positive correlation was observed between the NLR and the HY stage in OFF in the sPD cohort in both cohorts. For that reason, we further performed multivariate linear regression analyses to assess whether there were other variables confounding our results. After adjusting for age, sex, disease duration and LEDD, the correlation between the NLR and the HY stage did not remain statistically significant. In the HUVR cohort, the analysis showed that the NLR was not statistically significant (p=0.16), whereas age was a clear confounding factor (p<0.05) [F(5,119)=17.06, p<0.001]. Likewise, in the PPMI cohort, the analysis showed that the NLR was not statistically significant (p=0.50), whereas age was a clear confounding factor (p<0.05) [F(5,275)=3.62, p<0.001]. To conclude, no correlation was found the peripheral immune response evaluated in our study and motor impairment based on HY stage in patients with PD.

Supplementary Table 23. The correlation (Spearman) between motor impairment assessed by Hoehn & Yahr stage and the different blood cells counts as well as the NLR in patients with Parkinson's disease from the discovery and the PPMI replication cohorts

	**Hoehn y Yahr scale in OFF state**							
	Whole PD cohort		sPD		*LRRK2*-PD		*GBA*-PD	
	Discovery (n=222)	PPMI (n=401)	Discovery (n=132)	PPMI (n=281)	Discovery (n=44)	PPMI (n=66)	Discovery (n=46)	PPMI (n=54)
**Lymphocytes (x10^3 cells/µL)**	- 0.07 (0.30)	- 0.11 (0.04)*	- 0.11 (0.23)	-0.13 (0.03)*	- 0.02 (0.91)	- 0.02 (0.89)	- 0.08 (0.62)	- 0.19 (0.18)
**Neutrophils (x10^3 cells/µL)**	+ 0.09 (0.20)	+ 0.06 (0.22)	+ 0.16 (0.08)	+ 0.06 (0.29)	+ 0.05 (0.76)	- 0.04 (0.77)	- 0.07 (0.67)	+ 0.24 (0.09)
**Monocytes (x10^3 cells/µL)**	+ 0.05 (0.50)	+ 0.05 (0.35)	- 0.04 (0.67)	+ 0.06 (0.29)	+ 0.07 (0.66)	- 0.15 (0.29)	+ 0.17 (0.27)	+ 0.21 (0.13)
**NLR**	+ 0.11 (0.13)	+ 0.14 (0.006)*	+ 0.20 (0.03)*	+ 0.17 (0.006)*	+ 0.06 (0.71)	- 0.02 (0.88)	- 0.05 (0.75)	+ 0.30 (0.03)*

Note: values are expressed as rho correlation coefficient (p value). *A p value < 0.05 was considered statistically significant. Abbreviations: sPD, sporadic Parkinson's disease; *GBA*-PD, patients with *GBA*-associated Parkinson's disease; *LRRK2*-PD, patients with *LRRK2*-associated Parkinson's disease; n, number of subjects; NLR, neutrophil-to-lymphocyte ratio.

Eventually, we evaluated whether there was an association between the peripheral immune response and motor impairment based on MDS-UPDRS-III scale, which is a much more precise motor scale compared to HY stage. Data are shown in supplementary Table 24 below. In the Spearman's correlation analyses, the MDS-UPDRS-III score showed to be associated with the NLR in the whole cohort ($p<0.001$), and in the different PD cohorts. For that reason, further multivariate regression analyses were performed. The MDS-UPDRS-III remained positive correlated with the NLR ($p<0.05$), after adjusting per age, gender, disease duration and LEDD [$F(5,381)=6.22$, $p<0.001$)]. Moreover, multivariate linear regression analyses also showed the MDS-UPDRS-III was positively correlated with PD subgroups, regardless of their genetic background. Although it was only statistically significant in *LRRK2*-PD group ($p=0.04$), there was a trend to significance in sPD ($p=0.19$) and *GBA*-PD ($p=0.09$).

Supplementary Table 24. The correlation (Spearman) between motor impairment based on MDS-UPDRS-III scale and the different blood cells counts as well as the NLR in patients with Parkinson's disease from the PPMI replication cohort

	**MDS-UPDRS-III scale in OFF state**			
	Whole PD cohort (n=388)	sPD (n=281)	*LRRK2*-PD (n=56)	*GBA*-PD (n=51)
**Lymphocytes (x10^3 cells/µL)**	- 0.10 (0.06)	- 0.08 (0.21)	- 0.10 (0.45)	- 0.10 (0.49)
**Neutrophils (x10^3 cells/µL)**	+ 0.13 (0.01)*	+ 0.08 (0.20)	+ 0.26 (0.06)	+ 0.26 (0.07)
**Monocytes (x10^3 cells/µL)**	+ 0.09 (0.09)	+ 0.05 (0.41)	+ 0.19 (0.16)	0.15 (0.31)
**NLR**	+ 0.18 (<0.001)*	+ 0.12 (0.05)*	+ 0.32 (0.02)*	0.21 (0.13)

Note: values are expressed as rho correlation coefficient (p value). *A p value < 0.05 was considered statistically significant. Abbreviations: MDS-UPDRS-III, MDS Unified Parkinson's disease rating part III scale; n, number of subjects; sPD, sporadic Parkinson's disease; *GBA*-PD, patients with *GBA*-associated Parkinson's disease; *LRRK2*-PD, patients with *LRRK2*-associated Parkinson's disease; NLR: neutrophil-to-lymphocyte ratio.

c. Cognitive impairment.

Finally, Spearman's correlation analyses were performed to determine whether any of the blood cells counts or the NLR correlated to cognitive impairment assessed by the MOCA scale. Our discovery cohort was assessed retrospectively so MOCA scale was not available for all participants. These analyses could only be performed in the PPMI cohort. No correlation was found between MOCA score and either the different blood cells counts or the NLR in PPMI cohort.

Supplementary Table 25. The correlation (Spearman) between cognitive impairment based MOCA scale and the different blood cells counts as well as the NLR in patients with Parkinson's disease from the PPMI replication cohort.

	**MOCA score**			
	Whole PD cohort (n=400)	sPD (n=281)	*LRRK2*-PD (n=65)	*GBA*-PD (n=54)
**Lymphocytes (x10^3 cells/µL)**	- 0.02 (0.56)	+ 0.03 (0.66)	- 0.12 (0.32)	+ 0.04 (0.75)
**Neutrophil (x10^3 cells/µL)**	+ 0.03 (0.54)	+ 0.07 (0.27)	+ 0.06 (0.62)	- 0.22 (0.12)
**Monocytes (x10^3 cells/µL)**	+ 0.01 (0.90)	+ 0.04 (0.46)	- 0.05 (0.70)	- 0.22 (0.10)
**NLR**	+ 0.03 (0.56)	+ 0.04 (0.51)	+ 0.11 (0.37)	- 0.22 (0.12)

Note: values are expressed as rho correlation coefficient (p value). *A p value < 0.05 was considered statistically significant. Abbreviations: MOCA, Montreal Cognitive Assessment; PD, Parkinson's disease; sPD, sporadic Parkinson's disease; *GBA*-PD, patients with *GBA*-associated Parkinson's disease; *LRRK2*-PD, patients with *LRRK2*-associated Parkinson's disease; n, number of subjects; NLR, neutrophil-to-lymphocyte ratio.

Supplementary Information 2. Methods

The mutational screening of all *LRRK2* exons and intron–exon boundaries was performed using a High Resolution Melting (HRM) analysis and/or targeted resequencing. The gene regions which encode functional domains of *LRRK2* begin within exon 22 (amino acid 984), so, in this study, we used HRM analysis to screen for variations from that exon until exon 51. HRM reactions were performed on a LightCycler-480 (LC480) instrument, and HRM curve acquisition and analysis were performed using LC480 software version 1.3 (Roche Applied Science). All samples showing abnormal melting profiles were sequenced by Sanger sequencing. The list with the primers used in our study for *LRRK2* screening by HRM is shown below in Supplementary Table 26. Targeted resequencing was performed using a customized Haloplex Target Enrichment Panel (including *LRRK2*), which was designed using Agilent's online Sure Design tool, following the manufacturer's protocol (Agilent Technologies, Inc., Santa Clara, CA, USA). Samples were sequenced employing the Illumina NextSeq platform (Illumina Inc., San Diego, CA, USA). Filtered variants predicted as pathogenic were validated by Sanger sequencing. To determine the origin of each patient with R1441G mutation, haplotype analysis was performed by testing microsatellite markers and single-nucleotide polymorphisms spanning the gene region.

The procedure followed to do the *GBA* screening is outlined below. Twenty-five nanograms of genomic DNA was used for each polymerase chain reaction (PCR). To prevent amplification of the neighbouring pseudogene, *GBA* was first amplified in four large fragments that only and specifically amplified the functional gene but not the nearby pseudogene. PCR primer couples were designed based on the known genomic sequence (NG_009783.1). The list with the primers used in our study for *GBA* screening is shown below in Supplementary Table 27. For the mutational screening, we studied isoform 1 of the *GBA* gene (NM_001005741. 2), which contains 12 exons, including a noncoding exon 1. The mutational screening of all exons and intron-exon boundaries was then performed using a combination of HRM analysis and direct DNA resequencing. HRM reactions were performed on a LC480 instrument, and HRM curve acquisition and analysis were performed using LC480 software version 1.3 (Roche Applied Science, Indianapolis, IN, USA). Samples showing abnormal melting profiles, including those with variants, were sequenced on both strands using the BigDye terminator cycle sequencing kit (Applied Biosystems, Foster City, CA, USA) and resolved on an ABI3500 genetic analyzer (Applied Biosystems). We have adopted the conventional nomenclature, which refers to the processed protein and excludes the 39-residue signal peptide.

Supplementary Table 26. Complete list of primers used in *LRRK2* screening

Primer	Sequences
Exon 20 F2	AATTGTTGATTTCTAAGTTGCTGGT
Exon 20 R2	TGGGTCCTATTGTTCAATGTCAGT
Exon 21 F	GATTTTACAAAGGGAATGGACTGTG
Exon 21 R	GTCAGCAGCAAAACACAACATAATC
Exon 22 F	TGTCCTCTTCTCCAATAAATGACAG
Exon 22 R	TGGAGGAAATTCAACCAAACAC
Exon 23 F	GCTAGGAGGTGCTCACTAAACTTT
Exon 23R	AAGCTCTTCAAATGTCTTGAAAGTT
Exon 24 F	GCTAGACTTAAGTTCCTCAGATGG
Exon 24 R	TCAGCATATTTAGGCAACCC
Exon 25 F	AATGAGTCCTCTTTGATGCTGTTC
Exon 25 R	AAGGGTCCATATATGACTCATCTTG
Exon 26 F	CACTATTGGTAGCTGTTCTTATTTTTG
Exon 26R	AAGGTTCTGTTCCAGCTAATGTG
Exon 27 F	ACCCTGGGGAAAATTATTTGTG
Exon 27 R	GAAGCTTCTAGTTTCATGAAATTGG
Exon 28 F	CTTCCTTCCCACCAACAGG
Exon 28 R	TGTCCATCAAAGTCACAGAGAG
Exon 29 F	CAAATACTAGGTTTCTTCAACAGCG
Exon 29 R	CATACAGTCTACCAGGTTTCTGGAT
Exon 30 F	GGATTCTTGCCTGTCGTTTG
Exon 30 R	CTCGGAAAGTTTCCCAATTCAA
Exon 31 F	AGCAGGCCCAGTTTGAAAG
Exon 31 R	GACATTTCTAGGCAGTTGAGAATC
Exon 32 F	TTAGCACTGAATTTGCCAACC
Exon 32 R	CCGTATGGATATTCTCTCAACTTTG
Exon 33 F	AAAGCCCCTTGATATTTGTTC
Exon 33 R	ATGCTTTGACCATAACCCCC
Exon 34 F	TCTTTCTGACTACTTTCACTGAGCA
Exon 34 R	TTCTTTACCTGCTTGGAACCAG
Exon 35 F	AGGTTGGGTGTTTTGTGAGG
Exon 35 R	ATGCCATCTCCCTAATTTCTC
Exon 36 F	GGAAGCAGTTAATAATTAATGGCTC
Exon 36 R	AAACATTCAAATTGTTTCCTTACC
Exon 37 F	CTTTGCGACAGTATGAGGTTTAGA
Exon 37 R	TGAAGGATCACTTAAAAGCATTTGT
Exon 38 F	TTCACATCAAAACCACAAATTTATG

Supplementary Table 27. Complete list of primers used in *GBA* screening

**Primer**	**Sequences**
Exon 1A F	5’-GGAAGGTTAGGAATCCTCTGAGC
Exon 1A R	5’-GAAAAGCAGCCCTGGGGAGT
Exon 1B f	5’-GCCTCTGCATGAGTGACCGT
Exon 1B r	5’-TTAAGTGCGAACGCAGGGAG
Exon 2 F	5’-CCGGAATTACTTGCAGGGCT
Exon 2 R	5’-CCTGGATTCAAAGAGAGTCTGTCA
Exon 3 F2	5’-GTGGGCCTTGTCCTAATGAA
Exon 3 R2	5’-ACCACCTGCTTACTGGAAGG
Exon 4 F	5’-GCAGATGTGTCCATTCTCCA
Exon 4 R	5’-CACTGACACCATTTACCTCTAGGA
Exon 5 F	5’-TTCCCGCTGGGTACTGATAC
Exon 5 R	5’-CGAAAAGTTTCAATGGCTCT
Exon 6 F	5’-CAGGAGCCCAAGTTCCCTTT
Exon 6 R	5’-CTACAGTTTCTCAACCCCCAGA
Exon 7 F	5’-GTGGAGGCTAATGGCTGAAC
Exon 7 R3	5’-ACAGATCAGCATGGCTAAAT
Exon 8 F	5’-CACCCAGCTGGTCTGGTC
Exon 8 R	5’-GCTCTAAGTTTGGGAGCCAGT
Exon 9 F	5’-CTGTGTGCAAGGTCCAGGAT
Exon 9 R	5’-AGGTCTGAGGTCTGCTTTGC
Exon 10 F2	5’-CACAGCTGCCTCTCCCACAT
Exon 10 R4	5’-GTCACTTCCTGCCTCCATG
Exon 11 F	5’-GTCCGTGGGTGGGTGACT
Exon 11 R	5’-ACGCTGTCTTCAGCCCACT
Exon 12 F2	5’-TGGCAGGATCACACTCAG
Exon 12 R2	5’-TGCTGTGCCCTCTTTAGTCA
