# Supplementary material for: Peripheral inflammatory immune response differs among sporadic and familial Parkinson’s disease
Source: NPJ Parkinsons Dis. 2023 Jan 31;9:12. doi: 10.1038/s41531-023-00457-5 (PMC9889312; doi:10.1038/s41531-023-00457-5)
Supplement: Supplementary file 2 — Reporting Summary [file 41531_2023_457_MOESM2_ESM.pdf]

nature portfolio

Corresponding author(s): Pablo Mir and Pilar Gómez-Garre

Last updated by author(s): 2023-01-06

Reporting Summary

Nature Portfalio wishes to improve the reproducibility of the work that we publish. This farm provides structure far consistency and transparency in reporting. Far further infarmation on Nature Portfalio policies, see our Editorial Policies and the Editorial Policy Checklist.

Statistics

Far all statistical analyses, confirm that the fallowing items are present in the figure legend, table legend, main text, ar Methods section.

n/a	Confirmed	
	X	The exact sample size (n) far each experimental group/condition, given as a discrete number and unit of measurement
	X	A statement on whether measurements were taken from distinct samples ar whether the same sample was measured repeatedly
	X	The statistical test(s) used AND whether they are one- or two-sided<br>*Only common tests shou/d be described so/e/y by name; describe more complex techniques in the Methods section.*
	X	A description of all covariates tested
	X	A description of any assumptions ar corrections, such as tests of normality and adjustment far multiple comparisons
	X	A full description of the statistical parameters including central tendency (e.g. means) ar other basic estimates (e.g. regression coefficient) AND variation (e.g. standard deviation) ar associated estimates of uncertainty (e.g. confidence intervals)
☐	X	Far null hypothesis testing, the test statistic (e.g. *F, t,* r) with confidence intervals, effect sizes, degrees of freedom and *P* value noted<br>*Give P values as exact values whenever suitable.*
X		Far Bayesian analysis, infarmation on the choice of priors and Markov chain Monte Cario settings
X		Far hierarchical and complex designs, identification of the appropriate level far tests and full reporting of outcomes
X		Estimates of effect sizes (e.g. Cohen's *d,* Pearson's r), indicating how they were calculated

Our web col/ection on statistics for biologists contains artic/es on many ofthe points above.

Software and code

Policy infarmation about availability of computer code

Data collection	No software for data collection was used.
Data analysis	All statistical analyses were performed using the R version 3.5.1., and GraphPad Prism 8 software (GraphPad Software, Inc.).

Far manuscripts utilizing custom algorithms or software that are central to the research but not yet described in published literature, software must be made available to editors and reviewers. We strongly encourage code deposition in a community repository (e.g. GitHub). See the Nature Portfalio guidelines far submitting code & software far further infarmation.

Data

Policy infarmation about availability of data

All manuscripts must include a data availability statement. This statement should provide the fallowing infarmation, where applicable:

- Accession codes, unique identifiers, or web links for publicly available datasets
- A description of any restrictions on data availability
- For clinical datasets or third party data, please ensure that the statement adheres to our 2º.lkY,

Data availability: The data that support the findings of this study does not present restrictions and are available upon a request that is reasonable made and that can be provided by the corresponding author [P.M., P.G.-G.] due to participants privacy.

Human research participants

Policy infarmation about studies involving human research participants and Sex and Gender in Research.

Reporting on sex and gender

For assessing a possible sex influence of peripheral inflammatory immune response, Sex-stratified analyses were also performed in both cohorts. All data is shown in the results section and in supplementary information part B.

Population characteristics

This project was a retrospective study, including a discovery cohort with patients with Parkinson's disease (PD) from the Movement Disorder Clinic at Hospital Universitario Virgen del Rocio in Seville, Spain. PD was diagnosed following the Movement Disorder Society Clinical Diagnostic Criteria (Postuma et al. 2015). Patients with PD were classified into three subgroups according to their genetic background: 132 sPD patients, 44 LRRK2-PD patients and 46 GBA-PD patients. sPD patients did not have any variants in the PD-associated genes. The LRRK2-PD group included 33 LRRK2 p.G2019S PD patients and 11 LRRK2 p.R1441G PD patients. The list of GBA pathogenic variants considered for the inclusion of patients in the GBA-PD group is shown in Table 4.

Moreover, we also included a replication cohort. We used as replication cohort the Parkinson's Progression Markers Initiative (PPMI) cohort: an international, multisite, prospective, longitudinal cohort study. Details regarding the PPMI study have been published23 and are available on the PPMI website (http://www.ppmi-info.org). We applied the same criteria as for our discovery cohort. The replication study included 401 patients with PD [281 sPD patients, 66 LRRK2-PD patients (54 with p.G2019S and 12 with p.R1441G mutations), 54 GBA-PD patients] and a group of 174 HCs. The list of GBA pathogenic variants considered is shown in Table 4

Recruitment

For our discovery cohort, patients with PD were recruited from the Movement Disorder Clinic at Hospital Universitario Virgen del Rocio (Seville, Spain). Healthy controls were volunteers from the same geographical area, and they were not considered for the study if they had any neurodegenerative disorder, a family history of PD, or a variant in LRRK2 or GBA genes.
For validating the peripheral immune profile in PD according to their genetic background, we used as replication cohort the Parkinson's Progression Markers Initiative (PPMI) cohort: an international, multisite, prospective, longitudinal cohort study. The PPMI data used in this study were downloaded on June 2, 2022.

Ethics oversight

We obtained consent from the local ethics committee (Hospital Universitario Virgen del Rocío, Seville, Spain) in accordance with the Declaration of Helsinki, and written informed consent from all the participants in the study. The PPMI study was approved by the local institutional review boards of all participating sites.

Note that full information on the approval ofthe study protocol must also be provided in the manuscript.

Field-specific reporting

Please select the one below that is the best fit far your research. If you are not sure, read the appropriate sections befare making your selection.

[X] Lite sciences ■ Behavioural & social sciences ■ Ecological, evolutionary & environmental sciences

For a reference copy of the document with all sections, see nature.com/documents/nr-reporting-summary-flat.pdf

Life sciences study design

All studies must disclose on these points even when the disclosure is negative.

Sample size

This project was a retrospective study. No sample size calculation was performed. All patients with Parkinson's disease genetically characterized were included, and then selection criteria were applied. Total leukocyte count and subpopulations (neutrophils, lymphocytes, monocytes, eosinophils, and basophils) in peripheral blood were measured in the Central Laboratory of our center using Sysmex XN automated haematology analyser (at Hospital Universitario Virgen del Rocio, Seville, Spain).

Data exclusions

Data of patients with Parkinson's disease and data of healthy controls were excluded following the pre-established exclusion criteria. Also, data of those individuals with missing values were excluded too

Replication

A replication cohort was used for validating our results. The Parkinson's Progressive Markers Initiative cohort was used.

Randomization

No randomization was performed.

Blinding

Investigators were blinded during retrospective data collection (demographic and clinical data from both healthy controls and patients with Parkinson's disease) and analysis.

Reporting for specific materials, systems and methods

We require information from authors about sorne types of materials, experimental systems and methods used in many studies. Here, indicate whether each material, system or method listed is relevant to your study. If you are not sure if a list item applies to your research, read the appropriate section before selecting a response.

Materials & experimental systems		
n/a	Involved in the study	
X		Antibodies
X		Eukaryotic cell lines
X		Palaeontology and archaeology
X		Animals and other organisms
X		Clinical data
X		Dual use research of concern

Methods		
n/a	Involved in the study	
X		ChIP-seq
	X	Flow cytometry
X		MRI-based neuroimaging

Antibodies

Antibodies used	N/A
Validation	N/A

Eukaryotic cell lines

Policy information about cell lines and Sex and Gender in Research

Cell line source(s)	N/A
Mycoplasma contamination	
Authentication	N/A.
Commonly misidentified lines (See ICLAC register)	N/A.

Palaeontology and Archaeology

Specimen provenance	N/A
Specimen deposition	N/A.
Dating methods	N/A

■ Tick this box to confirm that the raw and calibrated dates are available in the paper or in Supplementary Information.

Ethics oversight	N/A.

Note that full information on the approval ofthe study protocol must also be provided in the manuscript.

Animals and other research organisms

Policy information about studies involving animals; ARRIVE guidelines recommended far reporting animal research, and Sex and Gender in Research

Laboratory animals	N/A.

Wild animals	*We state that the study did not involve wild animals.*
Reporting on sex	N/A
Field-collected samples	
Ethics oversight	

Note that full information on the approval of the study protocol must also be provided in the manuscript.

Clinical data

Policy information about clinical studies

All manuscripts should comply with the ICMJE guidelines far publication of clinical research and a completed CONSORT checklist must be included with ali submissions.

Clinical trial registration	N/A
Study protocol	N/A.
Data collection	N/A.
Outcomes	N/A.

Dual use research of concern

Policy information about dual use research of concern

Hazards

Could the accidental, deliberate or reckless misuse of agents or technologies generated in the work, or the application of information presented in the manuscript, pose a threat to:

No	Yes	
X		Public health
X		National security
X		Crops and/or livestock
X		Ecosystems
X		Any other significant area

Experiments of concern

Does the work involve any of these experiments of concern:

No	Yes	
X		Demonstrate how to render a vaccine ineffective
X		Confer resistance to therapeutically useful antibiotics or antiviral agents
X		Enhance the virulence of a pathogen or render a nonpathogen virulent
X		Increase transmissibility of a pathogen
X		Alter the host range of a pathogen
X		Enable evasion of diagnostic/detection modalities
X		Enable the weaponization of a biological agent or toxin
X		Any other potentially harmful combination of experiments and agents

ChIP-seq

Data deposition

■ Confirm that both raw and final processed data have been deposited in a public database such as GEO.

■ Confirm that you have deposited or provided access to graph files (e.g. BED files) for the called peaks.

Data access links<br>*May remain private before publication.*	*N/A.*
Files in database submission	*N/A*
Genome browser session<br>(e.g. UCSC)	*N/A*

Methodology

Replicates	*N/A.*
Sequencing depth	*N/A*
Antibodies	*N/A*
Peak calling parameters	*N/A*
Data quality	*N/A.*
Software	*N/A.*

Flow Cytometry

Plots

Confirm that:

■ The axis labeis state the marker and fluorochrome used (e.g. CD4-FITC).

■ The axis scales are clearly visible. Include numbers along axes only for bottom left plot of group (a 'group' is an analysis of identical markers).

■ All plots are contour plots with outliers or pseudocolor plots.

X A numerical value for number of cells or percentage (with statistics) is provided.

Methodology

Sample preparation	The source of the cells is peripheral blood.
Instrument	Sysmex XN automated haematology analyser
Software	Flowing Software v.2.5.1
Cell population abundance	Sysmex XN automated haematology analyser
Gating strategy	It uses a fluorescence flow cytometry with forward-scattered, side-scattered, and side-fluorescence lights for cells determination

■ Tick this box to confirm that a figure exemplifying the gating strategy is provided in the Supplementary Information.

Magnetic resonance imaging – Not used in our study
